# Supplementary material for: The cost-effectiveness of penicillin allergy testing: Evidence and gaps from a systematic review
Source: PLoS One. 2025 Dec 19;20(12):e0337131. doi: 10.1371/journal.pone.0337131 (PMC12716781; doi:10.1371/journal.pone.0337131)
Supplement: S2 File — (DOCX) [file pone.0337131.s002.docx]

S2 File. Search strategy

UPDATE Search Strategies

# Project Name: ALABAMA Economic Evaluation of penicillin allergy Date: 14 November 2023

## EconPapers (RePeC) all available dates

Search Date: 14 November 2023

**Free text search:9**  documents matched the search for ("beta lactam*" OR penicillin*) AND (allerg* OR hypersensitiv* OR intoler* OR anaphyl* OR delabel*)

## Embase Classic+Embase (Ovid) <1947 to 2023 November 13>

Search Date: 14 November 2023

1 health economics/ 40958

2 exp economic evaluation/ 358817

3 exp health care cost/ 346481

4 pharmacoeconomics/ or "drug cost"/ or drug utilization/ or "utilization review"/ 180077

5 socioeconomics/ and economics/ 16208

6 *socioeconomics/ 25447

7 Economic model/ 3315

8 *fee/ 6766

9 *"cost"/ 15117

10 cost*.ti. 198880

11 (cost* adj2 (effective* or utilit* or benefit* or minimi* or evaluat* or analy* or study or studies or consequenc* or compar* or efficienc* or variable* or unit or estimate*)).ab. 333154

12 (price or prices or pricing).tw. 73184

13 (economic* or pharmacoeconomic* or pharmaco-economic*).tw. 476035

14 budget*.tw. 48585

15 (value adj1 (money or monetary)).tw. 1138

16 (financ* adj2 (cost* or data or healthcare or "health care")).tw. 15582

17 financ*.tw. and economics/ 16184

18 (expenditure* not energy).tw. 52125

19 quality adjusted life year/ 35679

20 (eq-5d* or eq5d* or euroquol* or euroqol* or euroqual* or euro-quol* or euro-qol* or euro-qual*).tw. 31224

21 quality adjusted life.tw. 26669

22 (qaly or qalys or qald or qale or qtime).tw. 26880

23 disability adjusted life.tw. 6598

24 disability-adjusted life year/ 4735

25 (daly or dalys).tw. 6284

26 (SF6D or sf 6d or short form 6d or shortform6d).tw. 1847

27 health* year* equivalent*.tw. 41

28 (hye or hyes).tw. 181

29 health utilit*.tw. 4536

30 (hui1 or hui2 or hui3).tw. 654

31 disutil*.tw. 1279

32 standard gamble*.tw. 1200

33 Utility value*.tw. 4137

34 (time trade off or time tradeoff).tw. 2374

35 (hqol or h qol or hr qol or hrqol).tw. 39300

36 (pqol or qls).tw. 757

37 or/1-36 1479726

38 exp animals/ not (exp animals/ and exp humans/) 5956456

39 exp nonhuman/ not (exp nonhuman/ and exp human/) 5319972

40 exp experimental animal/ 861829

41 exp veterinary medicine/ 72244

42 animal experiment/ 3076442

43 ((energy or oxygen* or metaboli*) adj3 (expenditure* or cost*)).tw. 53005

44 or/38-43 8786412

45 37 not 44 1353691

46 *allergic reaction/ or exp *allergy/ or exp *drug hypersensitivity/ or anaphylaxis/ 208920

47 exp penicillin derivative/ 399389

48 exp beta lactam antibiotic/ 576923

49 46 and (47 or 48) 14580

50 penicillin allergy/ 3959

51 49 or 50 [MESH Penicillin allergy] 16449

52 (allerg* or hypersensitiv* or intoleran* or anaphyla*).tw. 543666

53 (penicillin* or beta-lactam*).tw. 147096

54 (amoxicillin or Amdinocillin or Cyclacillin or Methicillin or Nafcillin or Cloxacillin or Floxacillin or Dicloxacillin or Penicillanic Acid or Tazobactam or Ampicillin or Carbenicillin or Carfecillin or Sulbenicillin or Sulbactam or Ticarcillin).tw. [named penicillins] 128188

55 53 or 54 245551

56 52 and 55 [pen allergy] 11764

57 51 or 56 22130

58 45 and 57 [ec evaluations of penicillin allergy] 956

59 (delabel* or de-label* or mislabel* or Mis-label*).tw,kf. 3234

60 ((antibiotic* or antimicrobial*) adj stewardship).tw,kf. 15189

61 antimicrobial stewardship/ 10831

62 or/59-61 [delabelling terms] 20448

63 46 or 50 or 52 [hypersensitivity terms] 599904

64 62 and 63 [delabelling allergies] 1220

65 45 and 62 and 63 [ec eval delabelling allergies] 205

66 58 or 65 989

67 limit 66 to conference abstract 180

68 66 not 67 809

## International HTA Database (INAHTA) – all available dates

<https://database.inahta.org/>

Search date: 14 November 2023

All fields: (penicillin* OR beta-lactam*) AND (allerg* OR hypersensitiv* OR intoleran* OR anaphyla* OR delabel*) = 6*

*Note: 12 retrieved in 2022 update, all 6 above already in Endnote. Delabel* alone did not find any records*

## Ovid MEDLINE(R) ALL <1946 to November 10, 2023>

Search Date: 14 November 2023

1 Economics/ 27513

2 exp Economics, Dental/ 4074

3 exp Economics, Nursing/ 4013

4 exp Economics, Medical/ 14404

5 exp Economics, pharmaceutical/ 3113

6 exp Economics, Hospital/ 25763

7 exp "Costs and Cost Analysis"/ 267176

8 exp "Fees and Charges"/ 31420

9 exp budgets/ 14157

10 exp "Value of Life"/ec [Economics] 253

11 budget*.tw. 36295

12 cost*.ti. 146158

13 (cost* adj2 (effective* or utilit* or benefit* or minimi* or evaluat* or analy* or study or studies or consequenc* or compar* or efficienc* or variable* or unit or estimate*)).ab. 239324

14 (economic* or pharmacoeconomic* or pharmaco-economic*).tw. 376662

15 (price or prices or pricing).tw. 52366

16 (financ* adj2 (cost* or data or "health care" or healthcare)).tw. 11567

17 (fee or fees).tw. 21847

18 (value adj1 (money or monetary)).tw. 885

19 quality-adjusted life years/ 15921

20 (eq-5d* or eq5d* or euroquol* or euroqol* or euroqual* or euro-quol* or euro-qol* or euro-qual*).tw. 17384

21 exp models, economic/ 16242

22 markov chains/ 16047

23 quality adjusted life.tw. 17665

24 (qaly or qalys or qald or qale or qtime).tw. 14616

25 disability adjusted life.tw. 5524

26 (daly or dalys).tw. 4851

27 Disability-Adjusted Life Years/ or Healthy Life Expectancy/ [new 2022] 275

28 "Global Burden of Disease"/ [new 2017] 2044

29 health* year* equivalent*.tw. 40

30 (hye or hyes).tw. 75

31 (hui1 or hui2 or hui3).tw. 447

32 disutil*.tw. 649

33 standard gamble*.tw. 910

34 Utility value*.tw. 2271

35 (time trade off or time tradeoff).tw. 1660

36 (hqol or h qol or hrqol or hr qol).tw. 24361

37 (pqol or qls).tw. 469

38 (sf6d or sf 6d or short form 6d or shortform 6d or sf sixd or sf six d).tw. 1013

39 exp animals/ not (exp animals/ and exp humans/) 5169565

40 exp Veterinary Medicine/ 26242

41 exp Animal Experimentation/ 10379

42 ((energy or oxygen* or metaboli*) adj3 (expenditure* or cost*)).tw. 42571

43 or/39-42 5222761

44 or/1-38 966024

45 44 not 43 901973

46 exp penicillins/ or exp beta-Lactams/ 140281

47 exp Drug Hypersensitivity/ 49878

48 Hypersensitivity/ or Anaphylaxis/ or exp Hypersensitivity, Immediate/ or exp hypersensitivity, delayed/ 362781

49 46 and (47 or 48) [MESH penicillin allergy] 6189

50 (allerg* or hypersensitiv* or intoleran* or anaphyla*).tw. 348070

51 (penicillin* or beta-lactam*).tw. 105253

52 (amoxicillin or Amdinocillin or Cyclacillin or Methicillin or Nafcillin or Cloxacillin or Floxacillin or Dicloxacillin or Penicillanic Acid or Tazobactam or Ampicillin or Carbenicillin or Carfecillin or Sulbenicillin or Sulbactam or Ticarcillin).tw. [named penicillins] 93572

53 50 and (51 or 52) [penicillin allergy] 6006

54 49 or 53 9356

55 45 and 54 [Ec Eval penicillin allergy - all] 218

56 (delabel* or de-label* or mislabel* or Mis-label*).tw,kf. 1925

57 ((antibiotic* or antimicrobial*) adj stewardship).tw,kf. 10492

58 Antimicrobial Stewardship/ 3451

59 or/56-58 [delabelling terms] 12933

60 47 or 48 or 50 [hypersensitivity terms] 578114

61 59 and 60 [delabelling allergies] 582

62 45 and 61 [Ec eval - delabelling] 53

63 55 or 62 [Ec eval delabelling or penicillin allergies] 226

## Scopus (Elsevier B.V.) 1823 - Present

Search Date: 14 November 2023

15 ( ( TITLE-ABS-KEY ( allerg* OR hypersensitiv* OR intoleran* OR anaphyla* ) ) AND ( ( TITLE-ABS-KEY ( amoxicillin OR amdinocillin OR cyclacillin OR methicillin OR nafcillin OR cloxacillin OR floxacillin OR dicloxacillin OR "penicillanic acid" OR tazobactam OR ampicillin OR carbenicillin OR carfecillin OR sulbenicillin OR sulbactam OR ticarcillin ) ) OR ( TITLE-ABS-KEY ( penicillin* OR beta-lactam* ) ) OR ( TITLE-ABS-KEY ( delabel* OR "de-label*" OR mislabel* OR "Mis-label*" ) ) OR ( TITLE-ABS-KEY ( ( antibiotic* OR antimicrobial* ) W/1 stewardship ) ) ) ) AND ( ( TITLE-ABS-KEY ( budget* OR economic* OR pharmacoecon* OR "pharmaco-econ*" OR qaly OR qalys OR qald OR qale OR qtime OR hrqol OR eq-5d* OR eq5d* OR euroquol* OR euroqol* OR euroqual* OR euro-quol* OR euro-qol* OR euro-qual* OR price OR prices OR pricing OR fee OR fees ) ) OR ( TITLE-ABS-KEY ( markov OR "quality adjusted life" OR "disability adjusted life" OR daly OR dalys OR "health* year* equivalent*" OR hye OR hyes OR hui1 OR hui2 OR hui3 OR disutil* ) ) OR ( TITLE-ABS-KEY ( "standard gamble*" OR "utility value*" OR "time trade off" OR "time tradeoff" OR hqol OR "h qol" OR hrqol OR "hr qol" OR pqol OR qls OR sf6d OR "sf 6d" OR "short form 6d" OR "shortform 6d" OR "sf sixd" OR "sf six d" ) ) OR ( TITLE-ABS-KEY ( value W/1 ( money OR monetary ) ) ) OR ( TITLE-ABS-KEY ( financ* W/2 ( cost* OR data OR "health care" ) ) ) OR ( TITLE-ABS-KEY ( cost* W/2 ( effective* OR utilit* OR benefit* OR minimi* OR evaluat* OR analy* OR study OR studies OR consequenc* OR compar* OR efficienc* OR variable* OR unit OR estimate* ) ) ) OR ( TITLE ( "quality of life" OR qol ) ) ) ... **801 document results [#8 AND #14 Ec Eval pen allergy or delabelling]**

14 ( TITLE-ABS-KEY ( allerg* OR hypersensitiv* OR intoleran* OR anaphyla* ) ) AND ( ( TITLE-ABS-KEY ( amoxicillin OR amdinocillin OR cyclacillin OR methicillin OR nafcillin OR cloxacillin OR floxacillin OR dicloxacillin OR "penicillanic acid" OR tazobactam OR ampicillin OR carbenicillin OR carfecillin OR sulbenicillin OR sulbactam OR ticarcillin ) ) OR ( TITLE-ABS-KEY ( penicillin* OR beta-lactam* ) ) OR ( TITLE-ABS-KEY ( delabel* OR "de-label*" OR mislabel* OR "Mis-label*" ) ) OR ( TITLE-ABS-KEY ( ( antibiotic* OR antimicrobial* ) W/1 stewardship ) ) ) ... 22,373 document results #9 AND (#10 or #11 or #12 or #113)

13 TITLE-ABS-KEY ( ( antibiotic* OR antimicrobial* ) W/1 stewardship ) 13,847 document results

12 TITLE-ABS-KEY ( delabel* OR "de-label*" OR mislabel* OR "Mis-label*" ) 5,076 document results

11 TITLE-ABS-KEY ( penicillin* OR beta-lactam* ) 270,883 document results

10 TITLE-ABS-KEY ( amoxicillin OR amdinocillin OR cyclacillin OR methicillin OR nafcillin OR cloxacillin OR floxacillin OR dicloxacillin OR "penicillanic acid" OR tazobactam OR ampicillin OR carbenicillin OR carfecillin OR sulbenicillin OR sulbactam OR ticarcillin ) 313,949 document results

9 TITLE-ABS-KEY ( allerg* OR hypersensitiv* OR intoleran* OR anaphyla* ) 654,109 document results

8 ( TITLE-ABS-KEY ( budget* OR economic* OR pharmacoecon* OR "pharmaco-econ*" OR qaly OR qalys OR qald OR qale OR qtime OR hrqol OR eq-5d* OR eq5d* OR euroquol* OR euroqol* OR euroqual* OR euro-quol* OR euro-qol* OR euro-qual* OR price OR prices OR pricing OR fee OR fees ) ) OR ( TITLE-ABS-KEY ( markov OR "quality adjusted life" OR "disability adjusted life" OR daly OR dalys OR "health* year* equivalent*" OR hye OR hyes OR hui1 OR hui2 OR hui3 OR disutil* ) ) OR ( TITLE-ABS-KEY ( "standard gamble*" OR "utility value*" OR "time trade off" OR "time tradeoff" OR hqol OR "h qol" OR hrqol OR "hr qol" OR pqol OR qls OR sf6d OR "sf 6d" OR "short form 6d" OR "shortform 6d" OR "sf sixd" OR "sf six d" ) ) OR ( TITLE-ABS-KEY ( value W/1 ( money OR monetary ) ) ) OR ( TITLE-ABS-KEY ( financ* W/2 ( cost* OR data OR "health care" ) ) ) OR ( TITLE-ABS-KEY ( cost* W/2 ( effective* OR utilit* OR benefit* OR minimi* OR evaluat* OR analy* OR study OR studies OR consequenc* OR compar* OR efficienc* OR variable* OR unit OR estimate* ) ) ) OR ( TITLE ( "quality of life" OR qol ) ) 4,760,585 document results [ec eval /or #1-#7]

7 TITLE ( "quality of life" OR qol ) 113,598 document results

6 TITLE-ABS-KEY ( cost* W/2 ( effective* OR utilit* OR benefit* OR minimi* OR evaluat* OR analy* OR study OR studies OR consequenc* OR compar* OR efficienc* OR variable* OR unit OR estimate* ) ) 1,130,388 document results

5 TITLE-ABS-KEY ( financ* W/2 ( cost* OR data OR "health care" ) ) 66,755 document results

4 TITLE-ABS-KEY ( value W/1 ( money OR monetary ) ) 13,137 document results

3 TITLE-ABS-KEY ( "standard gamble*" OR "utility value*" OR "time trade off" OR "time tradeoff" OR hqol OR "h qol" OR hrqol OR "hr qol" OR pqol OR qls OR sf6d OR "sf 6d" OR "short form 6d" OR "shortform 6d" OR "sf sixd" OR "sf six d" ) 35,785 document results

2 TITLE-ABS-KEY ( markov OR "quality adjusted life" OR "disability adjusted life" OR daly OR dalys OR "health* year* equivalent*" OR hye OR hyes OR hui1 OR hui2 OR hui3 OR disutil* ) 269,491 document results

1 TITLE-ABS-KEY ( budget* OR economic* OR pharmacoecon* OR "pharmaco-econ*" OR qaly OR qalys OR qald OR qale OR qtime OR hrqol OR eq-5d* OR eq5d* OR euroquol* OR euroqol* OR euroqual* OR euro-quol* OR euro-qol* OR euro-qual* OR price OR prices OR pricing OR fee OR fees ) 3,604,941 document results

## Web of Science Core Collection: Citation Indexes

Search date: 14 November 2023

Simultaneous search of the following databases

- WOS.SCI: 1900 to 2023
- WOS.AHCI: 1975 to 2023
- WOS.ESCI: 2015 to 2023
- WOS.ISTP: 1990 to 2023
- WOS.SSCI: 1900 to 2023
- WOS.ISSHP: 1990 to 2023

*Data updated 2023-11-12*

1: (budget* or economic* or pharmacoecon* or "pharmaco-econ*" or qaly or qalys or qaly or qale or qiime or hrqol or eq-5d* or eq5d* or euroquol* or euroqol* or euroqual* or euro-quol* or euro-qol* or euro-qual* or price or prices or pricing or fee or fees or markov or "quality adjusted life" or "disability adjusted life" or daly or dalys or "health* year* equivalent*" or hye or hayes or hui3 or hui2 or hui3 or disutil* or "standard gamble*" or "utility value*" or "time trade off" or "time tradeoff" or hqol or "h qol" or hrqol or "hr qol" or pool or qls or sfed or "sf 6d" or "short form 6d" or "shortform 6d" or "sf sixd" or "sf six d") (Topic) Results: 2685237

2: value NEAR/1 (money or monetary) (Topic) Results: 8682

3: financ* NEAR/2 (cost* or data or "health care") (Topic) Results: 28331

4: cost* NEAR/2 (effective* or utilit* or benefit* or minimi* or evaluat* or analy* or study or studies or consequenc* or compar* or efficienc* or variable* or unit or estimate*) (Topic) Results: 664141

5: "quality of life" OR QOL (Title) Results: 135416

6: #5 OR #4 OR #3 OR #2 OR #1 Results: 3318430

7: allerg* or hypersensitiv* or intoleran* or anaphyla* (Topic) Results: 423580

8: penicillin* or beta-lactam* (Topic) Results: 116468

9: amoxicillin or Amdinocillin or Cyclacillin or Methicillin or Nafcillin or Cloxacillin or Floxacillin or Dicloxacillin or "Penicillanic Acid" or Tazobactam or Ampicillin or Carbenicillin or Carfecillin or Sulbenicillin or Sulbactam or Ticarcillin (Topic) Results: 97922

10: delabel* OR "de-label*" OR mislabel* OR "Mis-label*" (Topic) Results: 3469

11: ( antibiotic* OR antimicrobial* ) near/1 stewardship (Topic) Results: 11339

12: #11 OR #10 OR #9 OR #8 Results: 203575

13: #7 AND #6 AND #12 Results: 260
