# Supplementary material for: The cost-effectiveness of penicillin allergy testing: Evidence and gaps from a systematic review
Source: PLoS One. 2025 Dec 19;20(12):e0337131. doi: 10.1371/journal.pone.0337131 (PMC12716781; doi:10.1371/journal.pone.0337131)
Supplement: S3 File — (DOCX) [file pone.0337131.s003.docx]

S3 File. Studies excluded at full text screening stage with reasons

| No. | Reference | Reason for exclusion |
| --- | --- | --- |
| 1 | Askew et al. Cost-effectivenss of penicillin skin testing in GBS plus pregnant women with penicillin allergy | Publication type (abstract) |
| 2 | Sastre J, Manso L, Sanchez-García S, Fernández-Nieto M. Medical and economic impact of misdiagnosis of drug hypersensitivity in hospitalized patients. Journal of allergy and clinical immunology. 2012 Feb 1;129(2):566-7. | Population: mixed penicillin (38%) and other allergies (62%), without presenting results for penicillin allergy subgroup. |
| 3 | Al-Hasan MN, Acker EC, Kohn JE, Bookstaver PB, Justo JA. Impact of Penicillin Allergy on Empirical Carbapenem Use in Gram-Negative Bloodstream Infections: An Antimicrobial Stewardship Opportunity. Pharmacotherapy. 2018;38(1):42-50. | Intervention |
| 4 | Arroliga ME, Wagner W, Bobek MB, Hoffman-Hogg L, Gordon SM, Arroliga AC. A pilot study of penicillin skin testing in patients with a history of penicillin allergy admitted to a medical ICU. Chest. 2000;118(4):1106-8. | Outcome |
| 5 | Chisholm CA, Katz VL, McDonald TL, Bowes Jr WA. Penicillin desensitization in the treatment of syphilis during pregnancy. American Journal of Perinatology. 1997;14(9):553-7. | Intervention |
| 6 | Clark KE, Briand ME, Kapoor O, Pirasteh A. Impact of a Standardized Beta-Lactam Allergy Questionnaire on Aztreonam Use. Journal of Pharmacy Practice. 2019;32(4):399-403. | Intervention |
| 7 | Gugkaeva Z, Crago JS, Yasnogorodsky M. Next step in antibiotic stewardship: Pharmacist-provided penicillin allergy testing. Journal of Clinical Pharmacy and Therapeutics. 2017;42(4):509-12. | Publication type |
| 8 | Jones EP, Kim AS. Penicillin allergy testing: A strategic approach to increasing referrals from primary care physicians. Annals of Allergy, Asthma and Immunology. 2019;123(1):96-7. | Publication type |
| 9 | Kleris R, Tang M, Radojicic C, Lugar PL. Pricking away at penicillin allergy with a dedicated outpatient clinic. Journal of Allergy and Clinical Immunology: In Practice. 2019;7(4):1358-9.e1. | Publication type |
| 10 | Lee GC. CORR Insights: Is Vancomycin-only Prophylaxis for Patients With Penicillin Allergy Associated With Increased Risk of Infection After Arthroplasty? Clinical Orthopaedics and Related Research. 2016;474(7):1607-9. | Publication type |
| 11 | Lee RU, Banks TA, Waibel KH, Rodriguez RG. Penicillin Allergy...Maybe Not? The Military Relevance for Penicillin Testing and De-labeling. Mil Med. 2019;184(3):e163-e8. | Outcome |
| 12 | Parikh P, Patel NC, Trogen B, Feldman E, Meadows JA. The economic implications of penicillin allergy. Ann Allergy Asthma Immunol. 2020;6:06. | Publication type |
| 13 | Ramsey A, Staicu ML. Use of a Penicillin Allergy Screening Algorithm and Penicillin Skin Testing for Transitioning Hospitalized Patients to First-Line Antibiotic Therapy. Journal of Allergy and Clinical Immunology: In Practice. 2018;6(4):1349-55. | Outcome |
| 14 | Rubin R. Overdiagnosis of Penicillin Allergy Leads to Costly, Inappropriate Treatment. Jama. 2018;320(18):1846-8. | Publication type |
| 15 | Sacco KA, Bates A, Brigham TJ, Imam JS, Burton MC. Clinical outcomes following inpatient penicillin allergy testing: A systematic review and meta-analysis. Allergy. 2017;72(9):1288-96. | Study type |
| 16 | Sagar PS, Katelaris CH. Utility of penicillin allergy testing in patients presenting with a history of penicillin allergy. Asia Pac Allergy. 2013;3(2):115-9. | Outcome |
| 17 | Skibba N, Fischer J, Loecker B. Pilot program of pharmacist managed penicillin allergy skin testing on inpatients at a medical center to determine cost-benefit. Pharmacotherapy. 2014;34(6):E115-E. | Publication type |
| 18 | Temino VM, Gauthier TP, Lichtenberger P. Outpatient penicillin skin testing has greater value in targeted patient populations. Annals of Allergy, Asthma and Immunology. 2018;120(4):441-2. | Publication type |
| 19 | Wang LA, Patel K, Kuruvilla ME, Shih J. Direct amoxicillin challenge without preliminary skin testing for pediatric patients with penicillin allergy labels. Annals of Allergy, Asthma and Immunology. 2020;125(2):226-8. | Publication type |
| 20 | Wolfson AR, Huebner EM, Blumenthal KG. Acute care beta-lactam allergy pathways: approaches and outcomes. Annals of Allergy, Asthma and Immunology. 2019;123(1):16-34. | Study type |
| 21 | Au LYC, Siu AM, Yamamoto LG. Cost and Risk Analysis of Lifelong Penicillin Allergy. Clin Pediatr (Phila). 2019;58(11):1309-14. | Intervention |
| 22 | Collins CD, Scheidel C, Anam K, Polega S, Malani AN, Hayward A, et al. Impact of an antibiotic side chain-based cross-reactivity chart combined with enhanced allergy assessment processes for surgical prophylaxis antimicrobials in patients with beta-lactam allergies. Clinical infectious diseases : an official publication of the Infectious Diseases Society of America. 2020;10. | Intervention |
| 23 | Estep PM, Ferreira JA, Dupree LH, Aldridge PJ, Jankowski CA. Impact of an antimicrobial stewardship initiative to evaluate beta-lactam allergy in patients ordered aztreonam. Am J Health-Syst Pharm. 2016;73(5):S8-13. | Intervention |
| 24 | Huang K-HG, Cluzet V, Hamilton K, Fadugba O. The Impact of Reported Beta-Lactam Allergy in Hospitalized Patients With Hematologic Malignancies Requiring Antibiotics. Clinical Infectious Diseases. 2018;67(1):27-33. | Intervention |
| 25 | Irawati L, Hughes JD, Keen NJ, Golledge CL, Joyce AW. Influence of penicillin allergy on antibiotic prescribing patterns and costs. Journal of Pharmacy Practice and Research. 2006;36(4):286-90. | Intervention |
| 26 | Kraemer MJ, Caprye-Boos H, Berman HS. Increased use of medical services and antibiotics by children who claim a prior penicillin sensitivity. Western Journal of Medicine. 1987;146(6):697-700. | Intervention |
|  | Li M, Krishna MT, Razaq S, Pillay D. A real-time prospective evaluation of clinical pharmaco-economic impact of diagnostic label of 'penicillin allergy' in a UK teaching hospital. J Clin Pathol. 2014;67(12):1088-92. | Intervention |
| 27 | MacLaughlin EJ, Saseen JJ, Malone DC. Costs of beta-lactam allergies: selection and costs of antibiotics for patients with a reported beta-lactam allergy. Arch Fam Med. 2000;9(8):722-6. | Intervention |
| 28 | Picard M, Begin P, Bouchard H, Cloutier J, Lacombe-Barrios J, Paradis J, et al. Treatment of Patients with a History of Penicillin Allergy in a Large Tertiary-Care Academic Hospital. Journal of Allergy and Clinical Immunology: In Practice. 2013;1(3):252-7. | Intervention |
| 29 | Powell N, Honeyford K, Sandoe J. Impact of penicillin allergy records on antibiotic costs and length of hospital stay: a single-centre observational retrospective cohort. J Hosp Infect. 2020;106(1):35-42. | Intervention |
| 30 | Sade K, Holtzer I, Levo Y, Kivity S. Economic burden and outcome of a positive history of penicillin allergy in patients seen in a tertiary medical center. Journal of Allergy and Clinical Immunology. 2000;105(1):S341-S. | Intervention |
| 31 | Sade K, Holtzer I, Levo Y, Kivity S. The economic burden of antibiotic treatment of penicillin-allergic patients in internal medicine wards of a general tertiary care hospital. Clin Exp Allergy. 2003;33(4):501-6. | Intervention |
| 32 | Sousa-Pinto B, Cardoso-Fernandes A, Araujo L, Fonseca JA, Freitas A, Delgado L. Clinical and economic burden of hospitalizations with registration of penicillin allergy. Ann Allergy Asthma Immunol. 2018;120(2):190-4.e2. | Intervention |
| 33 | Yoon K, Lee M, Patel R, Park Z. Successful Implementation of a Simple Algorithm to Manage Penicillin Allergy in an Acute Care Community Hospital. Annals of Pharmacotherapy. 2018;52(6):603-4. | Intervention |
| 34 | Campbell S, Hauler G, Immler EL, Seiti S, Dandache P, Srinivas P. #1840: Pharmacist-led Penicillin Allergy Assessment in the Emergency Department Reduced Empiric Fluoroquinolone Use. Clinical Infectious Diseases. 2020;71:E506-E8. | Outcome |
| 35 | Fésüs A, Benkő R, Matuz M, Kungler-Gorácz O, Fésüs MÁ, Bazsó T, et al. #2669: The effect of pharmacist-led intervention on surgical antibacterial prophylaxis (SAP) at an orthopedic unit. Antibiotics. 2021;10(12). | Intervention |
| 36 | Harper HM, Sanchez M. #1796: Review of Pharmacist Driven Penicillin Allergy Assessments and Skin Testing: A Multi-Center Case-Series. Hospital Pharmacy. 2021. | Intervention |
| 37 | Mody D, Burke C, Minson Q. #1781: Short and long term impact of combining restrictive and enabling interventions to reduce aztreonam consumption in a community hospital. International Journal of Clinical Pharmacy. 2021;43:1345-51. | Outcome |
| 38 | Bodega-Azuara J, Belles Medall MD, Edo-Peñarrocha J, et al. Eur J Hosp Pharm Epub ahead of print: [please include Day Month Year].  doi:10.1136/ ejhpharm-2022-003304 | Outcome |
| 39 | Cao H, Phe K, Laine GA, Russo HR, Putney KS, Tam VH. An institutional review of antimicrobial stewardship interventions. Journal of global antimicrobial resistance. 2016 Sep 1;6:75-7. | Intervention |
| 40 | Sobrino M, Muñoz-Bellido FJ, Macías E, Lázaro-Sastre M, de Arriba-Méndez S, Dávila I. A prospective study of costs associated with the evaluation of β-lactam allergy in children. The Journal of Pediatrics. 2020 Aug 1;223:108-13. | Duplicate |
| 41 | Dong Y, Zembles TN, Nimmer M, Brousseau DC, Vyles D. A potential cost savings analysis of a penicillin de-labeling program. Frontiers in Allergy. 2023 Mar 30;4:1101321. | Intervention |
| 42 | Gorsline CA, Afghan AK, Stone Jr CA, Phillips EJ, Satyanarayana G. Safety and value of pretransplant antibiotic allergy delabeling in a quaternary transplant center. Transplant Infectious Disease. 2022 Oct;24(5):e13885. | Population, Intervention, Study design |
| 43 | Liu MY, Challa M, McCoul ED, Chen PG. Economic viability of penicillin allergy testing to avoid improper clindamycin surgical prophylaxis. The Laryngoscope. 2023 May;133(5):1086-91. | Wrong study type, review using economic data from Sousa-Pinto et al. 2021 and unit costs of Blumenthal et al. 2018b |
| 44 | Yoo M, Madaras-Kelly K, Nevers M, Fleming-Dutra KE, Hersh AL, Ying J, Haaland B, Samore M, Nelson RE. A Veterans’ Healthcare Administration (VHA) antibiotic stewardship intervention to improve outpatient antibiotic use for acute respiratory infections: A cost-effectiveness analysis. Infection Control & Hospital Epidemiology. 2022 Oct;43(10):1389-95. | Intervention |
