# Supplementary material for: The cost-effectiveness of penicillin allergy testing: Evidence and gaps from a systematic review
Source: PLoS One. 2025 Dec 19;20(12):e0337131. doi: 10.1371/journal.pone.0337131 (PMC12716781; doi:10.1371/journal.pone.0337131)
Supplement: S1 Table — (DOCX) [file pone.0337131.s005.docx]

S1 Table. Cost elements measured in reviewed studies

| - Cost element | Testing strategy | Ab use | Adverse Effects | Length of hospital stay | Healthcare use subsequent episodes | Ab use –subsequent episodes |
| --- | --- | --- | --- | --- | --- | --- |
| 1. Allen et al. 2021 | ✓ |  |  |  |  |  |
| 2. Blumenthal et al. 2018b | ✓ |  |  |  |  |  |
| 3. Borch et al. 2006 |  | ✓ |  |  |  |  |
| 4. Bragg et al. 2023 |  |  |  |  |  |  |
| 5a. Brusco et al. 2023 (1) | ✓ |  |  |  |  |  |
| 5b. Brusco et al 2023 (2) | ✓ | ✓ |  | ✓ |  |  |
| 6. Chen et al. 2018 | ✓ | ✓ |  |  |  |  |
| 7. Dodek et al. 1999 | ✓ | ✓ | ✓ | ? |  |  |
| 8. Du Plessis et al. 2019 |  | ✓ |  | + |  | ✓ |
| 9. Englert et al. 2019 | x | x |  | + |  |  |
| 10. Fan et al. 2020 |  | ✓^1^ |  |  |  |  |
| 11. Ferre-Ybarz et al. 2015 | ✓ |  |  |  |  |  |
| 12. Foolad et al. 2019 | ✓ | ✓ |  |  |  |  |
| 13. Forrest et al. 2001 | ✓ | ✓ |  |  |  |  |
| 14. Harmon et al. 2020 | x | ✓ |  |  |  |  |
| 15. Heil et al. 2016 |  | x |  |  |  |  |
| 16. Jaoui et al. 2019 | ✓^2^ |  |  |  |  |  |
| 17. Jones and Bland 2017 |  | ✓ |  |  |  |  |
| 18. Jones et al. 2019 | x | ✓ |  |  |  |  |
| 19. King et al. 2016 | x | ✓ |  |  |  |  |
| 20. Lee et al. 2021 | ✓ |  |  |  | ✓ |  |
| 21. Li et al. 2019 |  | ✓ |  | ✓ | + |  |
| 22. Macy et al. 2017 | ✓ | + |  | ✓ | ✓ |  |
| 23. Macy et al. 1998 |  | ✓ |  |  | + | + |
| 24. Mattingly et al. 2019 | ✓ | ✓ | ✓ | ? | ✓ | ✓ |
| 25. Modi et al. 2019 | x | x |  | + |  |  |
| 26. Pagani et al. 2021 | ✓^3^ | ✓ | ✓ |  |  |  |
| 27. Phillips et al. 2000^4^ | ✓ | ✓ | x | ✓ |  |  |
| 28. Ramsey et al. 2020 | ✓ | ✓ |  |  |  |  |
| 29. Rimawi et al. 2013 |  | ✓ |  |  |  |  |
| 30. Staicu et al 2018 | x | ✓ |  |  |  |  |
| 31. Sousa-Pinto et al. 2021 | ✓ | ✓ | ✓ | ✓ | ✓ |  |
| 32. Sobrino et al. 2020 | ✓ |  |  |  |  |  |
| 33. Sobrino et al. 2021 | ✓ |  |  |  |  |  |
| 34. Thao et al. 2023 |  |  |  |  |  |  |
| 35. Vyles et al. 2018 |  |  |  |  |  | ✓ |

^1^ Includes extended outpatient regimens ^2^ Measured cost of testing and Inpatient vs outpatient visit ^3^ Used threshold analysis to identify breakeven point of intervention costs being offset by cost savings ^4^ Study did not measure costs for treating AEs, only their consequences in terms of LOS. The study did measure AEs as part of the outcome. + Study measured but did not value this item. X Study reported a cost but did not account for all relevant costs for this item.
