# Supplementary material for: The cost-effectiveness of penicillin allergy testing: Evidence and gaps from a systematic review
Source: PLoS One. 2025 Dec 19;20(12):e0337131. doi: 10.1371/journal.pone.0337131 (PMC12716781; doi:10.1371/journal.pone.0337131)
Supplement: S2 Table — (DOCX) [file pone.0337131.s006.docx]

Table S2. Data extraction of test costs.

| **Authors** | **Date of Publication** | **N** | Cost of test (per patient, USD) | Cost of comparator test (per patient, USD) | Population | Test type and setting | Data extractor (date) | Confirmed inclusion of outcome |
| --- | --- | --- | --- | --- | --- | --- | --- | --- |
| 1. Allen et al. | 2021 | 99 | 800.25 | 2073.29 | Paediatric | OC, non-allergist OP; comparator: day ward | DK, RMM 16/03/2025 | Yes |
| 2. Blumenthal et al. 2018b | 2018 | 30 | 287.02 | 109.59 | Outpatient | ST+OC OP; comparator: OC OP allergist | DK July 2023, RMM16/03/2025 | Yes |
| 4. Bragg et al. 2023 | 2019 | - | NA | NA | N/A | NA | DK, RMM 16/03/2025 | No, break even value |
| 5a. Brusco et al. 2023 (1) | 2017 | 16 | 204.55 | 171.57 | Outpatient | ST+OC OP; comparator OC OP (specialist) | DK, RMM 16/03/2025 | Yes |
| Brusco et al. 2023 (1) | 2023 | 200 | 29.77 | 17.02 | Inpatient | non-allergy specialist, OC;  comparator OC/DD | DK, RMM 16/03/2025 | No; omits costs of oral history |
| 6. Chen et al | 2018 | 58 | 287.02 | N/A | Inpatient | SPT+OC, non-allergist | DK, RMM 16/03/2025 | Yes |
| 7. Dodek et al. 1999 | 1999 | NA | 178.79 | NA | Inpatient | SPT | DK, RMM 16/03/2025 | No; figure not reported |
| 9. Englert et al. 2019 | 2018 | 80 | NA | NA | Outpatient | SPT+IDT | DK, RMM 16/03/2025 | No, only measured supplies |
| 11. Ferre-Ybarz et al. 2015 | 2006 | 100 | 288.74 | 187.96 | Outpatient | SPT; Comparator: DPT, allergist | DK, RMM 16/03/2025 | Yes |
| 12. Foolad et al. 2019 | 2019 | NA | NA | NA | Inpatients | SPT | DK, RMM 16/03/2025 | No, cites Blumenthal et al 2018 study |
| 13. Forrest et al. 2001 | 2001 | 95 | NA | NA | Inpatients | SPT+DPC | DK, RMM 16/03/2025 | No, not reported |
| 14. Harmon et al. 2020 | 2020 | 31 | NA | NA | Inpatients | SPT+DPC, pharmacist | RMM 16/03/2025 | No, not measured (charges for PST supplies) |
| 16. Jaoui et al. 2019 | 2019 | 456 | 70.57 | NA | Outpatient | DPC, allergist | DK, RMM 16/03/2025 | Yes |
| 18. Jones et al. 2019 | 2019 | 100 | 238.29 | NA | Outpatients | SPT+DPC | DK, RMM 16/03/2025 | No, includes drug supply charges only |
| 19. King et al. 2016 | 2016 | 50 | NA | NA | Inpatients | SPT+DPC | DK, RMM 16/03/2025 | No, not measured |
| 20. Lee et al. 2021 | 2021 | NA | NA | NA | Inpatients | SPT | RMM 16/03/2025 | No, cites another source |
| 22. Macy et al. 2017 | 2017 | 308 | NA | NA | Outpatient | SPT+DPC | DK, RMM 16/03/2025 | No, not reported other than cost of reagents and nursing time. |
| 24. Mattingly et al. 2019 | 2019 | NA | NA | NA | Inpatient | SPT | DK, RMM 16/03/2025 | Not reported, cites another study |
| 25. Modi et al. 2019 | 2019 | NA | NA | NA | Inpatient | SPT+DPC | DK, RMM 16/03/2025 | No, not measured |
| 26. Pagani et al. 2021 | 2021 | NA | NA | NA | Inpatient | SPT+DPC | DK, RMM 15/03/2025 | No, break even analysis |
| 27. Phillips et al. 2000^4^ | 2000 | NA | NA | NA | Inpatient | SPT | DK, RMM 15/03/2025 | No, not reported other than cost of reagents and nursing time. |
| 28. Ramsey et al. 2020 | 2020 | 52 | 514.38 | 252.73 | Inpatient | SPT+DPC; comparator DPC, allergist | EK, RMM 15/03/2025 | Yes |
| 30. Staicu et al 2018 | 2018 | 50 | NA | NA | Outpatient | SPT+DPC | DK, RMM 15/03/2025 | No, not measured |
| 31. Sousa-Pinto et al. 2021 | 2021 | NA | NA | NA | Inpatients, Outpatient | SPT | DK, RMM 16/03/2025 | No, cites 9 other sources, including Blumenthal et al 2018, Chen et al. 2018; Ferre-Ibarz et al. 2015 |
| 32. Sobrino et al. 2020 | 2020 | 40 | 164.32 | NA | Outpatient | SPT+DPC | DK, RMM 16/03/2025 | Yes |
| 33. Sobrino et al. 2021 | 2021 | 296 | 185.08 | NA | Outpatient | SPT+DPC | DK, RMM 16/03/2025 | Yes |

NA: not reported.
