# Supplementary material for: The cost-effectiveness of penicillin allergy testing: Evidence and gaps from a systematic review
Source: PLoS One. 2025 Dec 19;20(12):e0337131. doi: 10.1371/journal.pone.0337131 (PMC12716781; doi:10.1371/journal.pone.0337131)
Supplement: S3 Table — (DOCX) [file pone.0337131.s007.docx]

Table S3. Data extraction of antibiotic costs outcome.

| **Authors** | **Date of Publication** | **N** | Cost of antibiotic before intervention (per patient, USD) | Cost of antibiotic after intervention (per patient, USD) | Cost difference (per patient, USD) | Time point | Data extractor (date) | Confirmed inclusion of outcome |
| --- | --- | --- | --- | --- | --- | --- | --- | --- |
| Fan et al | 2020 | 22 | NA | NA | 1.45 | Index hospital admission | DK, RMM 16/03/2025 | No, cost avoidance study; critical risk of bias |
| Ramsey et al | 2020 | 100 | NA | NA | 286.53 | Index hospital admission | DK July 2023, RMM16/03/2025 | No, cost avoidance study; critical risk of bias |
| Harmon et al | 2020 | 31 | NA | NA | 466.39 | Index hospital admission | DK, RMM 16/03/2025 | No, cost avoidance study; critical risk of bias |
| Modi et al | 2019 | 208 | NA | NA | N/A | Index hospital admission | DK, RMM 16/03/2025 | No, incomplete measure of outcome |
| Jones et al | 2017 | 36 | NA | NA | 277.59 | Duration of Ab therapy | DK, RMM 16/03/2025 | No, cost avoidance study; critical risk of bias |
| Foolad et al | 2019 | 33 | NA | NA | 1476.90 | Duration of Ab therapy | DK, RMM 16/03/2025 | No, cost avoidance study; critical risk of bias |
| Vyles et al | 2018 | 81 | 50.80 | 29.22 | 21.58 | 1-year | DK, RMM 16/03/2025 | Yes |
| Chen et al | 2018 | 91 | 1651.43 | 772.45 | 878.97 | Index hospital admission | DK, RMM 16/03/2025 | Yes |
| Staicu et al | 2018 | 50 | NA | NA | 587.79 | 2-4 weeks | DK, RMM 16/03/2025 | No, cost avoidance study; critical risk of bias |
| du Plessis et al | 2018 | 250 | 195.45 | 99.26 | 41.85 | Index hospital admission | DK, RMM 16/03/2025 | Yes |
| Rimawi et al | 2013 | 146 | 1065.15 | 758.56 | 306.59 | Index hospital admission | DK, RMM 16/03/2025 | No, cost avoidance study; critical risk of bias |
| Borch et al | 2006 | 43 | 550.57 | 235.69 | 314.87 | Index hospital admission (until end of test) | DK, RMM 16/03/2025 | No, cost avoidance study; critical risk of bias |
| Forrest et al | 2001 | 159 | 363.35 | 292.71 | 70.64 | Index hospital admission | DK, RMM 16/03/2025 | Yes |
| Macy | 1998 | 236 | 149.95 | 101.49 | 48.46 | 1 -year | DK, RMM 16/03/2025 | Yes |
| Macy et al. | 2017 | 308 | NA | NA | NA | 1-year | RMM 16/03/2025 | No, measured but did not evaluate outcome |
| Li et al | 2019 | 70 | 392.66 | 134.66 | 258.00 | Index hospital admission | DK, RMM 16/03/2025 | Yes |
| Englebert | 2019 | 22 | NA | 96.37 | NA | Index hospital admission | DK, RMM 16/03/2025 | No; non-comparative study |
| Brusco | 2020 | 218 | 135.76 | 58.15 | 77.61 | Index hospital admission | DK, RMM 16/03/2025 | Yes |
| du Plessis et al | 2018 | 250 | 329.41 | 109.75 | 219.65 | 1-year | DK, RMM 16/03/2025 | Yes |
| King et al | 2016 | 50 | 545.65 | 128.06 | 417.59 | Duration of AB therapy | DK, RMM 16/03/2025 | No, cost avoidance study; critical risk of bias |

NA: not reported.
