# Supplementary material for: The cost-effectiveness of penicillin allergy testing: Evidence and gaps from a systematic review
Source: PLoS One. 2025 Dec 19;20(12):e0337131. doi: 10.1371/journal.pone.0337131 (PMC12716781; doi:10.1371/journal.pone.0337131)
Supplement: S4 Table — (DOCX) [file pone.0337131.s008.docx]

Table S4. Data extraction of hospital costs outcome.

| **Authors** | **Date of Publication** | **N** | Mean cost of antibiotic before intervention (per patient, USD) | Mean cost of antibiotic after intervention (per patient, USD) | Mean Cost difference (per patient, USD) | Time point | Data extractor (date) | Confirmed inclusion of outcome |
| --- | --- | --- | --- | --- | --- | --- | --- | --- |
| Modi et al | 2019 | 208 | NA | N/A | N/A | Index hospital admission | DK, RMM 16/03/2025 | No, measured but did not evaluate this outcome |
| du Plessis et al | 2018 | 250 | NA | NA | N/A | Index hospital admission | DK, RMM 16/03/2025 | No, measured but did not evaluate outcome |
| Macy | 1998 | 236 | NA | NA | N/A | 1 -year, hospital days | DK, RMM 16/03/2025 | No, measured but did not evaluate outcome |
| Macy et al | 2017 | 308 | NA | NA | -8002.97 | 1 -year (avg. 3.6 yrs follow-up), hospital days | RMM 16/03/2025 | Yes; N tested patients; 1251 controls (p<0.001) |
| Macy et al. | 2017 | 308 | NA | NA | -748.69 | 1 -year (avg. 3.6 yrs follow-up), ED | RMM 16/03/2025 | Yes; N tested patients; 1251 controls (p=0.029) |
| Macy et al. | 2017 | 308 | NA | NA | -59.36 | 1-year (avg. 3.6 yrs follow-up), OP visits | RMM 16/03/2025 | Yes; N tested patients; 1251 controls (p<0.001) |
| Li et al | 2019 | 70 | 20305.29 | 15703.09 | -4602.20 | Index hospital admission | DK, RMM 16/03/2025 | Yes; (p<0.05) |
| Li et al | 2019 | 70 | NA | NA | -4602.20 | Hospital readmission | DK, RMM 16/03/2025 | No, measured but did not evaluate outcome |
| Englert | 2019 | 22 | NA | NA | NA | Index hospital admission | DK, RMM 16/03/2025 | No; non-comparative study; measured but did not evaluate outcome |
| Brusco | 2020 | 218 | 16937.96 | 8925.53 | -8012.43  (-13050, -2975) | Index hospital admission | DK, RMM 16/03/2025 | Yes, reported 95% CI in brackets. |

ED: emergency department; OP: outpatient; NA: not reported.
