# Supplementary material for: The cost-effectiveness of penicillin allergy testing: Evidence and gaps from a systematic review
Source: PLoS One. 2025 Dec 19;20(12):e0337131. doi: 10.1371/journal.pone.0337131 (PMC12716781; doi:10.1371/journal.pone.0337131)
Supplement: Attachment — Submitted filename: reviewed_PONE-D-24-34463.pdf [file pone.0337131.s010.pdf]

PLOS ONE

# The cost-effectiveness of penicillin allergy testing: a systematic review

--Manuscript Draft--

| | |
|---|---|
| **Manuscript Number:** | PONE-D-24-34463 |
| **Article Type:** | Research Article |
| **Full Title:** | The cost-effectiveness of penicillin allergy testing: a systematic review |
| **Short Title:** | Cost-effective penicillin allergy testing |
| **Corresponding Author:** | Ruben E Mujica-Mota, BSc, MSc, PhD<br>University of Leeds<br>Leeds, UNITED KINGDOM OF GREAT BRITAIN AND NORTHERN IRELAND |
| **Keywords:** | Systematic review, penicillin allergy, testing, delabelling, costs, cost-effectiveness |
| **Abstract:** | Introduction<br><br>Patients with a penicillin allergy label (PAL) use more and broader-spectrum antibiotics, have worse health outcomes and cost more to treat than patients without a PAL. A significant proportion of penicillin allergy labels are incorrect; here we review the published evidence on the costs, health-related quality of life, and cost-effectiveness of penicillin allergy testing.<br><br>Methods<br><br>We conducted a systematic review of published economic studies of penicillin allergy testing. We searched Medline, Embase, Scopus, Web of Science, EconPapers (RePeC) and the International HTA Database (INAHTA) and included reports of economic evaluations of costs and/or health benefits of penicillin allergy testing strategies. The review followed a narrative synthesis.<br><br>Results<br><br>Thirty-five studies met the inclusion criteria. Most studies analysed the effect of testing on the costs of antibiotic use among patients admitted to hospital with a PAL. The median cost of skin testing plus oral challenge across five primary costing studies was USD 268 (range: 153, 480), which contrasts with the USD 367-591 range of antibiotic cost savings during the initial hospital admission. The median reduction of costs of antibiotic therapy was 57% (range: 52-66; 3 cohort studies with concurrent controls) and costs of length of hospital stay of initial admission 35% (range: 23-47, two prospective studies). Two studies presented evidence that penicillin allergy testing is cost-saving in an outpatient setting over 3.5-4.5 years. One model-based study reported that testing in inpatient settings is cost-saving. No reports on the effect of penicillin allergy testing on health-related quality of life were found.<br><br>Conclusions<br><br>The available evidence is insufficient to determine whether the reduction in antibiotic costs with penicillin-allergy testing is sufficient to offset the costs of testing. Further research is needed to measure the effects of inpatient and outpatient testing for penicillin allergy on healthcare use |
| **Order of Authors:** | Ruben E Mujica-Mota, BSc, MSc, PhD |
| | Miaoqing Yang, PhD |
| | Natalie King, BSc, Msc |
| | Shadia Ahmed |
| | Neil Powell |
| | Sue Pavitt |
| | Bethany Shinkins |

Powered by Editorial Manager® and ProduXion Manager® from Aries Systems Corporation

| | |
|---|---|
| | Jonathan Sandoe |
| **Additional Information:** | |
| **Question** | **Response** |
| **Financial Disclosure**<br><br>Enter a financial disclosure statement that describes the sources of funding for the work included in this submission. Review the submission guidelines for detailed requirements. View published research articles from *PLOS ONE* for specific examples.<br><br>This statement is required for submission and **will appear in the published article** if the submission is accepted. Please make sure it is accurate.<br><br>**Funded studies**<br>Enter a statement with the following details:<br>• Initials of the authors who received each award<br>• Grant numbers awarded to each author<br>• The full name of each funder<br>• URL of each funder website<br>• Did the sponsors or funders play any role in the study design, data collection and analysis, decision to publish, or preparation of the manuscript?<br><br>Did you receive funding for this work? | Yes |
| Please add funding details.<br>as follow-up to "**Financial Disclosure**<br><br>Enter a financial disclosure statement that describes the sources of funding for the work included in this submission. Review the submission guidelines for detailed requirements. View published research articles from *PLOS ONE* for specific examples.<br><br>This statement is required for submission and **will appear in the published article** if the submission is accepted. Please make sure it is accurate. | NIHR PGfAR RP-PG-1214-20007 |

| | |
|---|---|
| **Funded studies**<br>Enter a statement with the following details:<br>• Initials of the authors who received each award<br>• Grant numbers awarded to each author<br>• The full name of each funder<br>• URL of each funder website<br>• Did the sponsors or funders play any role in the study design, data collection and analysis, decision to publish, or preparation of the manuscript?<br><br>Did you receive funding for this work?" | |
| Please select the country of your main research funder (please select carefully as in some cases this is used in fee calculation).<br>as follow-up to "**Financial Disclosure**<br><br>Enter a financial disclosure statement that describes the sources of funding for the work included in this submission. Review the submission guidelines for detailed requirements. View published research articles from *PLOS ONE* for specific examples.<br><br>This statement is required for submission and **will appear in the published article** if the submission is accepted. Please make sure it is accurate.<br><br>**Funded studies**<br>Enter a statement with the following details:<br>• Initials of the authors who received each award<br>• Grant numbers awarded to each author<br>• The full name of each funder<br>• URL of each funder website<br>• Did the sponsors or funders play any role in the study design, data collection and analysis, decision to publish, or preparation of the manuscript?<br><br>Did you receive funding for this work?" | UNITED KINGDOM - GB |
| **Competing Interests** | The authors have declared that no competing interests exist. |

| | |
|---|---|
| Use the instructions below to enter a competing interest statement for this submission. On behalf of all authors, disclose any competing interests that could be perceived to bias this work—acknowledging all financial support and any other relevant financial or non-financial competing interests.<br><br>This statement is required for submission and **will appear in the published article** if the submission is accepted. Please make sure it is accurate and that any funding sources listed in your Funding Information later in the submission form are also declared in your Financial Disclosure statement.<br><br>View published research articles from *PLOS ONE* for specific examples.<br><br>**NO authors have competing interests**<br><br>Enter: *The authors have declared that no competing interests exist.*<br><br>**Authors with competing interests**<br><br>Enter competing interest details beginning with this statement:<br><br>*I have read the journal's policy and the authors of this manuscript have the following competing interests: [insert competing interests here]*<br><br>* typeset | |
| **Ethics Statement**<br><br>Enter an ethics statement for this submission. This statement is required if the study involved:<br><br>• Human participants<br>• Human specimens or tissue<br>• Vertebrate animals or cephalopods<br>• Vertebrate embryos or tissues | N/A |

| | |
|---|---|
| • Field research<br><br>Write "N/A" if the submission does not require an ethics statement.<br><br>General guidance is provided below. Consult the submission guidelines for detailed instructions. **Make sure that all information entered here is included in the Methods section of the manuscript.** | |

| | |
|---|---|
| **Format for specific study types**<br><br>**Human Subject Research (involving human participants and/or tissue)**<br>• Give the name of the institutional review board or ethics committee that approved the study<br>• Include the approval number and/or a statement indicating approval of this research<br>• Indicate the form of consent obtained (written/oral) or the reason that consent was not obtained (e.g. the data were analyzed anonymously)<br><br>**Animal Research (involving vertebrate animals, embryos or tissues)**<br>• Provide the name of the Institutional Animal Care and Use Committee (IACUC) or other relevant ethics board that reviewed the study protocol, and indicate whether they approved this research or granted a formal waiver of ethical approval<br>• Include an approval number if one was obtained<br>• If the study involved *non-human primates*, add *additional details* about animal welfare and steps taken to ameliorate suffering<br>• If anesthesia, euthanasia, or any kind of animal sacrifice is part of the study, include briefly which substances and/or methods were applied<br><br>**Field Research**<br><br>Include the following details if this study involves the collection of plant, animal, or other materials from a natural setting:<br>• Field permit number<br>• Name of the institution or relevant body that granted permission | |
| **Data Availability**<br><br>Authors are required to make all data underlying the findings described fully available, without restriction, and from the time of publication. PLOS allows rare exceptions to address legal and ethical concerns. See the PLOS Data Policy and FAQ for detailed information. | Yes - all data are fully available without restriction |

| | |
|---|---|
| A Data Availability Statement describing where the data can be found is required at submission. Your answers to this question constitute the Data Availability Statement and **will be published in the article**, if accepted.<br><br>**Important:** Stating 'data available on request from the author' is not sufficient. If your data are only available upon request, select 'No' for the first question and explain your exceptional situation in the text box.<br><br>Do the authors confirm that all data underlying the findings described in their manuscript are fully available without restriction? | |
| **Describe where the data may be found in full sentences. If you are copying our sample text, replace any instances of XXX with the appropriate details.**<br><br>• If the data are **held or will be held in a public repository**, include URLs, accession numbers or DOIs. If this information will only be available after acceptance, indicate this by ticking the box below. For example: *All XXX files are available from the XXX database (accession number(s) XXX, XXX.).*<br>• If the data are all contained **within the manuscript and/or Supporting Information files**, enter the following: *All relevant data are within the manuscript and its Supporting Information files.*<br>• If neither of these applies but you are able to provide **details of access elsewhere**, with or without limitations, please do so. For example:<br><br>*Data cannot be shared publicly because of [XXX]. Data are available from the XXX Institutional Data Access / Ethics Committee (contact via XXX) for researchers who meet the criteria for access to confidential data.*<br><br>*The data underlying the results presented in the study are available from (include the name of the third party* | The detail data extracted from the studies included in the review are available from the authors upon request |

| | |
|---|---|
| *and contact information or URL).*<br>• This text is appropriate if the data are owned by a third party and authors do not have permission to share the data.<br><br>* typeset | |
| Additional data availability information: | |

Manuscript

Click here to access/download;Manuscript;PAL delabelling_main_body_numbers.docx

# The cost-effectiveness of penicillin allergy testing: a systematic review

Authors: Mujica-Mota R[1], Yang M[1], King N[1], Ahmed S[2], Powell N[3], Pavitt S[4], Shinkins B[5], Sandoe J[6].

1. Academic Unit of Health Economics, Leeds Institute of Health Sciences, University of Leeds, UK

2. Leeds Institute of Medical Research, School of Medicine, University of Leeds and Leeds Teaching Hospitals NHS Trust

3. Pharmacy Department, Royal Cornwall Hospital, Truro, United Kingdom TR1 3LJ / School of Biomedical Sciences, University of Plymouth, Plymouth, United Kingdom.

4. Dental Translational and Clinical Research Unit, University of Leeds, Leeds, UK

5. University of Warwick, Division of Health Sciences, Coventry, United Kingdom

6. Leeds Institute of Medical Research, University of Leeds, Leeds, United Kingdom.

Sponsor: University of Leeds Teaching Hospital NHS Foundation Trust

Funder: NIHR PGfAR RP-PG-1214-20007

Word count = 4260; Tables = 5; Figures = 1; Supplementary Files = 2

Address for correspondence:

Associate Professor Ruben Mujica-Mota

Academic Unit of Health Economics

Leeds Institute of Health Sciences

University of Leeds

Leeds, LS2 9LH

Email: R.E.Mujica-Mota@leeds.ac.uk

Contributions:

DK screened studies, extracted and analysed data studies and contributed to write up. MY contributed to screening, data extraction and write up. NK conducted the bibliographic searches, data management, and contributed to write up. RMM contributed to screening, data extraction and analysis, led the write up and is the guarantor of this study. NP contributed to screening and write up. SA contributed to screening and write up. BS designed the study, contributed to screening, data extraction and write up. JS contributed to the study design and write up.

COI:

RMM is supported in part by the National Institute for Health and Care Research (NIHR) Leeds Biomedical Research Centre (BRC) (NIHR203331). The views expressed are those of the author(s) and not necessarily those of the NHS, the NIHR or the Department of Health and Social Care.

**Abstract**

**Introduction**

Patients with a penicillin allergy label (PAL) use more and broader-spectrum antibiotics, have worse health outcomes and cost more to treat than patients without a PAL. A significant proportion of penicillin allergy labels are incorrect; here we review the published evidence on the costs, health-related quality of life, and cost-effectiveness of penicillin allergy testing.

**Methods**

We conducted a systematic review of published economic studies of penicillin allergy testing. We searched Medline, Embase, Scopus, Web of Science, EconPapers (RePeC) and the International HTA Database (INAHTA) and included reports of economic evaluations of costs and/or health benefits of penicillin allergy testing strategies. The review followed a narrative synthesis.

**Results**

Thirty-five studies met the inclusion criteria. Most studies analysed the effect of testing on the costs of antibiotic use among patients admitted to hospital with a PAL. The median cost of skin testing plus oral challenge across five primary costing studies was USD 268 (range: 153, 480), which contrasts with the USD 367-591 range of antibiotic cost savings during the initial hospital admission. The median reduction of costs of antibiotic therapy was 57% (range: 52-66; 3 cohort studies with concurrent controls) and costs of length of hospital stay of initial admission 35% (range: 23-47, two prospective studies). Two studies presented evidence that penicillin allergy testing is cost-saving in an outpatient setting over 3.5-4.5 years. One model-based study

reported that testing in inpatient settings is cost-saving. No reports on the effect of penicillin allergy testing on health-related quality of life were found.

## Conclusions

The available evidence is insufficient to determine whether the reduction in antibiotic costs with penicillin-allergy testing is sufficient to offset the costs of testing. Further research is needed to measure the effects of inpatient and outpatient testing for penicillin allergy on healthcare use

## Introduction

Penicillin and other beta-lactam antibiotics are the most frequent cause of medication induced anaphylaxis [1, 2, 3] and 6% of people in England have a record of penicillin allergy. However, at least 9 out of 10 people who believe they are penicillin-allergic are found not to be when tested, and so could safely take penicillins. These patients may be receiving less effective antibiotic treatments with additional long-term health risks. Penicillin-allergy records (or label) drive prescribing towards alternative broad-spectrum antimicrobials that contribute to increased antimicrobial resistance (AMR) and may result in poorer patient outcomes. Research has found that macrolide, tetracycline, cephalosporin and quinolone prescribing were all more common in patients with a record of penicillin allergy, compared to those without, and that antimicrobial prescriptions were almost twice as frequent in patients with a penicillin allergy label [4, 5].

Antibiotic allergies, including penicillin allergy, have been associated with suboptimal antibiotic therapy, increased antimicrobial resistance, increased length of stay, increased antibiotic-related adverse effects such as *Clostridioides difficile* infection, intensive care unit (ICU) admission, death, as well as increased treatment cost [6]. Antibiotic regimens deviate from the standard of care in approximately 40% of patients who report a penicillin allergy [7, 8]. The costs of the consequences of sub-optimal antibiotic therapy due to reported penicillin allergy are likely significant, reaching far beyond the actual differences in antibiotic costs [9].

The standard assessment for penicillin allergy involves skin testing followed by intradermal tests and oral drug provocation testing (oral challenge test). Several recent

studies have reported safe and effective testing for low-risk penicillin allergy labels using a direct oral challenge in children and increasingly in adults [10, 11, 12, 13]. Due to the prevalence of penicillin allergy labels and the limited capacity of existing specialist allergy clinics, there is increasing interest in expanding access to penicillin allergy testing through provision of non-allergy specialist delivered testing for low-risk patients. Generating evidence on the costs and benefits of different models of penicillin allergy testing services would help inform policy decisions and service design and planning.

The aim of this study was to review the published evidence on the cost-effectiveness of penicillin allergy testing and costs associated with a penicillin allergy label. This evidence is intended to inform sustainable service delivery models for increasing access to penicillin allergy testing and de-labelling of non-allergic patients.

## Methods

This systematic review was conducted according to the Cochrane Collaboration for Reviews and Centre for Reviews and Dissemination (CRD) guidelines [13, 14], and reported in line with the Preferred Reporting Items for Systematic Reviews and Meta-Analyses" (PRISMA) 2020 [15] (S1 File). The review protocol is registered in PROSPERO (CRD42021231848).

The objective of the systematic review was to identify and synthesise evidence from published studies on the healthcare costs, clinical consequences, and health-related quality of life associated with a penicillin allergy label and the cost-effectiveness of testing approaches for de-labelling individuals with an incorrect penicillin allergy label.

### Eligibility criteria

Studies were included in the review if they met the following criteria: i) study participants were patients with a penicillin allergy label or a self-reported penicillin allergy label; ii) participants underwent penicillin allergy de-labelling using any test; iii) one or more comparator groups were formed with participants receiving another test or no test; iv) measured both costs and health outcomes (full economic evaluation) or either costs or quality of life outcomes (partial economic evaluation). Only peer-reviewed publications written in English were included in the review.

We excluded conference abstracts, short notes, comments, editorials and study protocols. Reviews of the literature that did not synthesise findings into new estimates of cost effectiveness were excluded, although their reference lists were scrutinised for any studies not identified through screening of individual bibliographic

records. Studies not reporting on people who had undergone penicillin allergy testing were not included in the review.

Outcomes

Reported outcomes of interest were healthcare resource use, medical costs, and health-related quality of life for patients with a penicillin allergy label and patients who have had a penicillin allergy label removed. In addition, reports on cost-effectiveness of interventions for safely de-labelling individuals with an incorrect penicillin allergy label were reviewed.

Search Strategy

The searches were originally conducted in November 2020, then rerun in full on 14th November 2023 in the following electronic databases: Medline, Embase, Scopus, Web of Science, EconPapers (RePeC) and the International HTA Database (INAHTA).

The literature searches were designed using a combination of text words and database subject headings for two search concepts, penicillin allergy and economic evaluations. The search terms included terms and synonyms for: ‘penicillin’ OR ‘beta lactams’ AND ‘allergy’ AND a sensitive in-house search strategy developed to find cost and economic evaluation studies (S2 File). The searches were not limited by date or language and were peer-reviewed by a second Information Specialist using the PRESS checklist [16].

Study Selection

Two independent reviewers initially screened the title and abstract of each identified record. The full texts of those deemed potentially relevant were then reviewed against the eligibility criteria to identify studies for inclusion. All screening was conducted in Rayyan [17]. Disagreements were resolved by discussion.

### Data Extraction

Study characteristics were extracted from each included study, including publication details, the type of study conducted, the population included in the study, the intervention and comparators. Any results reported on health outcomes and cost measures, total or incremental costs were also extracted.

### Quality and risk of bias assessment

In a slight deviation from the protocol, we evaluated the methodological quality of the included economic evaluations using a published checklist [18] instead of the CHEERS statement [19], which covers many details beyond the scope of the studies in this area. Each study was appraised independently by two reviewers, with conflicts resolved by discussion.

### Data synthesis

The narrative synthesis was structured according to study setting (primary or community, hospital outpatient and inpatient, other), study design (prospective, retrospective or model based), and study type (costing study, cost of illness, cost comparison/minimisation analysis, cost effectiveness analysis). We also recorded whether the study was 1) a cohort study with concurrent or retrospective controls, 2)

a before-and-after study of provider organisation policy change or implementation, or 3) an individual counterfactual analysis of case series.

Cost estimates were presented in 2022 USD, using the latest purchasing power parities [20] and the US consumer price index [21] for converting original published figures reported in different years and currencies.

Intervention effect on healthcare cost estimates from primary data reported by more than one independent study were converted to percentage change units and summarised in terms of minimum, median and maximum values. Effect estimates for health-related quality of life and health outcomes from multiple independent primary studies were summarised in their original units using the same statistics.

## Results

Identified records and included studies

The search produced 2,117 records, with 1,312 unique records after de-duplication. Screening the title and abstracts led to the exclusion of 1,227 records, with 79 records included for full-text screening. This yielded 35 unique articles reporting on 36 studies meeting the final inclusion criteria. Four review articles were identified [22, 23, 24, 25]; we hand searched the references lists for these reviews but no additional articles were identified (Figure 1; see S3 File for list of articles excluded at the full-text screening stage with reasons for exclusion).

Fig 1. Study selection

Of the 36 included studies, 28 were observational studies and 8 were model-based evaluations. The majority of the observational studies were cost impact analyses of two or more testing strategies (n=17), followed by studies limited to measuring the costs of testing (n=6), and cost of therapy ('cost of illness') studies (n=5 studies). Four of the modelling studies were cost-effectiveness studies comparing costs and health outcomes of two or more testing strategies, with the remaining four comparing only costs. The descriptive summary characteristics of studies are presented in S1 Table and S2 Table, and their reported cost measures in S3 Table.

Study quality

The median score across the 36 studies was 9 (range: 3-14) out of 19 items (Table 1). Whilst most studies clearly described the population, the testing strategies under comparison and their study question, 50% of studies adopted a time horizon that was

too limited to capture relevant costs and health outcomes to their study question. Only 11% and 44% of studies adequately measured health outcomes and costs respectively, and 67% clearly presented an analysis of costs and/or benefit differences. In 75% of studies, no sensitivity analyses of assumptions used in measuring or analysing costs or benefits were presented, and 36% of studies did not discuss the generalisability of their findings beyond their local study setting. Almost two-thirds of studies reported conflicts of interest or presented no information on whether such conflict existed (Table 1).

Table 1. Study Quality: CHEC checklist [18]

| Item | % studies (N=36) meeting the item criteria |
|---|---|
| 1. Is the study population clearly described? | 100 |
| 2. Are competing alternatives clearly described? | 89 |
| 3. Is a well-defined research question posed in answerable form? | 97 |
| 4. Is the economic study design appropriate to the stated objective? | 72 |
| 5. Is the chosen time horizon appropriate in order to include relevant costs and consequences? | 50 |
| 6. Is the actual perspective chosen appropriate? | 69 |
| 7. Are all important and relevant costs for each alternative identified? | 44 |
| 8. Are all costs measured appropriately in physical units? | 39 |
| 9. Are costs valued appropriately? | 58 |
| 10. Are all important and relevant outcomes for each alternative identified? | 11 |
| 11. Are all outcomes measured appropriately? | 8 |
| 12. Are outcomes valued appropriately? | 0 |
| 13. Is an incremental analysis of costs and outcomes of alternatives performed? | 67 |
| 14. Are all future costs and outcomes discounted appropriately? | 19 |
| 15. Are all important variables, whose values are uncertain, appropriately subjected to sensitivity analysis? | 25 |
| 16. Do the conclusions follow from the data reported? | 75 |
| 17. Does the study discuss the generalizability of the results to other settings and patient/client groups? | 64 |
| 18. Does the article indicate that there is no potential conflict of interest of study researcher(s) and funder(s)? | 39 |
| 19. Are ethical and distributional issues discussed appropriately? | 3 |

## Costs measured in the studies

### *Costs of evaluating Beta-lactam allergy*

Nine studies reported on the costs of testing, of which six evaluated skin tests with oral challenge (ST+ OC) [26, 27, 28, 29, 30, 31] (apart from two [27, 31], these studies used intradermal injection after negative skin prick testing), and six evaluated direct drug provocation (DPC) testing [26, 27, 30, 32, 33, 34]. The median cost of ST with oral challenge at an outpatient allergy clinic was USD 268 (range: 153, 480), whilst DPC in an outpatient allergy clinic was USD139 (range: 66, 236). A prospective study in children reported that the cost of DPC delivered by a nurse and supervised by a consultant amounted to USD 747 [32]. A second report of DPC delivered by non-allergy specialists reported costs of USD 160 for low-risk patients tested by trained nursing, pharmacy or medical staff in Australia [33] (Table 2).

Table 2. Direct cost of PAL testing

| Testing strategy | Number of studies | Cost per patient (USD*) | | | Comments and sources |
|---|---|---|---|---|---|
| | | Median | Minimum | Maximum | |
| Skin Test (outpatient) | 1 | 177.90 | 177.90 | 177.90 | Trained nurse, pharmacist or medical staff [33]; skin prick and intradermal testing |
| Sink Test (inpatient) | 1 | 423.08 | 423.08 | 423.08 | Nurse, pharmacist or medical staff [33]; skin prick and intradermal testing |
| Skin test plus oral challenge (outpatient) | 5 | 267.95 | 153.40 | 480.19 | Allergist [26, 27, 28, 29, 30]; one was a retrospective study [26]; skin prick test + IDT, with one exception that did not use IDT [28][a] |
| Skin test plus oral challenge (inpatient) | 1 | 267.95 | 267.95 | 267.95 | Pharmacist [31] (skin prick test without IDT) |
| Oral Challenge (non-allergy specialists) | 2 | 453.44 | 159.82 | 747.07 | Outpatient. Trained nurse, pharmacist or medical staff [33]; consultant-supervised nurse [32][b] |

| | | | | | |
|---|---|---|---|---|---|
| Oral Challenge (allergist) | 4 | 138.89 | 65.88 | 235.94 | Outpatient [26, 27, 34, 30]; two retrospective [26, 34][c] |

* At year 2022 prices. [a] No study reported measure of sampling uncertainty [b] No study reported measure of sampling uncertainty. [c] No study reported measure of sampling uncertainty.

Three studies investigated patient travel and work absenteeism costs associated with seeking and receiving testing, the costs of which were as large as those of testing itself to the health system [28, 29, 33]. Two of these studies also reported variation in costs according to test result, which was negligible. They also reported a comparison between patients with immediate and patients with delayed reactions, with the latter being 50% larger than the former in adults [28] and negligible in children [29].

Low-risk penicillin allergy de-labelling in an inpatient setting was found to cost less than attending an outpatient clinic following discharge from hospital, whether only the direct healthcare costs or also patient and carer travel costs were included in the analysis [33]. This was due to the high proportion of patients missing their de-labelling appointments (11%), and having separate visits for assessment and testing (17%) in the outpatient setting, whereas inpatients had their allergy assessment, testing and communication of results within the index admission without needing further clinical appointments.

*Costs of antibiotic medication use*

Antibiotic costs were the most commonly investigated outcome (reported in 20 of the included studies), with all studies documenting cost savings [30, 31, 33, 35, 36, 37, 38, 39, 40, 41, 42, 43, 44, 45, 46, 47, 48, 49, 50, 51]. The ability to compare outcomes is low as studies varied widely in their methodology. In cohort studies with concurrent controls [33, 45, 46], the median reduction of antibiotic costs associated with testing

was 57% (range: 52,66), with the largest reduction reported in inpatients over 6 months of follow-up after discharge and the minimum in outpatients over a 1-year follow-up period (Table 3). Three prospective studies reported a median cost savings of USD 367 (range: 457,592) [30, 38, 41].

Table 3. Effect on healthcare costs of PAL testing

| Testing strategy | Number of studies | % reduction in cost per patient | | | Comments and sources |
|---|---|---|---|---|---|
| | | Median cost | Minimum | Maximum | |
| Antibiotic use (initial therapy) | 3 | 57 | 52 | 66 | Median is from prospective study of inpatients until hospital discharge [33]. Minimum is from an outpatient cohort over a 1 year follow up period after testing [46]; maximum is from a study of inpatients over 6 months of follow-up after discharge [45]. |
| Hospital LOS (Initial hospital admission) | 2 | 35 | 23 | 47 | Median: mid-point value of minimum and maximum. Minimum is from study report that DPC or SPT + OC reduced LOS by 23% among adult patients admitted with a diagnosis of type B Penicillin allergy that required penicillin-containing antibiotics (p<0.05 [45]; maximum is from study report that DPC among low risk admitted adults reduced such costs by 47% (p=0.002 [33]. |

In contrast, uncontrolled studies of ‘potential impact’ compared the observed antibiotic costs in a group of inpatients undergoing penicillin allergy testing with the counterfactual costs that would have been observed if those patients had not had their PAL tested [35, 44, 48, 49, 50]. The median reported projected reduction in antibiotic costs with skin testing plus oral challenge in the inpatient setting [35, 44, 48] was 57% (range: 29, 83).

As for antibiotic use in the community, one study reported antibiotic cost savings of USD 1368 associated with removing a PAL in 100 low-risk children attending the emergency department over a median follow-up of 1 year [50]. A second study compared the total number of antibiotic courses in the year before with the year after intervention in 250 patients at the imputed cost of the preferred prescribed antibiotic, which resulted in an antibiotic cost per day per patient that was 2.5 times greater in confirmed penicillin allergic patients than de-labelled patients [36].

*Costs of healthcare resource utilisation*

Four studies measured the impact of penicillin allergy de-labelling on hospital length of stay (LOS), three were limited to initial admission LOS [33, 37, 47] and one looked at LOS for admissions up to 6 months after testing [45]; two of these reported sufficient information to estimate the effect on costs [33, 45]. Adult inpatients with a type B penicillin allergy diagnosis requiring penicillin antibiotic treatment who were tested with DPC or ST + OC had a reduced LOS (23%, $p<0.05$) [45], whilst Brusco et al. found that the costs of admission (emergency department and acute including antibiotics) were reduced by 47% ($p=0.002$) among admitted adults who were low-risk and had a DPC [33]. One of the studies that presented no cost data found no statistically

detectable differences in LOS during index hospitalisation nor in length of ICU stay [47].

In a retrospective study of insurance claim records, individuals with a penicillin allergy history attending an outpatient allergy consultation for skin testing plus an oral challenge were subsequently observed to experience 0.55 fewer days in hospital and 0.09 fewer outpatient visits than matched controls annually over 3.6 years [52]. These effects on healthcare service use were estimated to amount to cost savings several orders of magnitude larger than the costs of the evaluated nurse-led penicillin allergy testing intervention [52]. In an economic model of outpatient penicillin allergy testing [53], downstream cost savings offset testing costs, and were driven by de-labelling, resulting in 3.05 (95% CI: 2.3, 3.8) fewer annual primary care visits over 4.5 years. This value was obtained from a published estimate of the excess number of primary care visits in an observational study of a group of patients with a PAL relative to matched controls without a PAL [54]. The estimated minimum de-labelling rate for skin testing with oral challenge to be cost-saving was 30-60% for inpatient and 10-50% for outpatient testing settings, and larger than for DPC (15-30% and 5-30%, respectively [53]).

Health outcomes

Two studies reported health outcomes [45, 47]. In a retrospective study of adults undergoing inpatient hematopoietic stem cell transplantation in a single centre, no detectable difference in 90-day post-transplant incidence of *Clostridioides difficile* nor mortality was observed (11% before and 7%, p=0.34, in both outcomes) after mandatory implementation of ST+OC [47]. In a prospective case series study of 70

SC+OC tested adult inpatients requiring penicillin, their rate of 6-month hospital readmission was 43% versus 61% in matched controls ($p<0.05$), and the rate of newly acquired multi-resistant organisms was 4 and 7% ($p=0.50$), respectively [45].

Health-related Quality of life

No study was found that reported evidence on the health-related quality of life of individuals with a PAL. Only a couple of studies, both based on decision tree models, reported results in terms of quality-adjusted life years (QALYs). One of them elicited health state utility values of treatment cure and failure in *Staphylococcus aureus* endocarditis under skin testing with and without adverse reaction to penicillin or toxicity from second-line therapy (vancomycin), and under no testing with and without vancomycin toxicity, from a nurse providing care for such inpatients [55]. The other was a study of skin testing in *S. aureus* bacteremia which obtained values for a 'post-septic episode with no other issue', 'disutility for adverse event/adverse drug reaction' independent of antimicrobial regimen and 'disutility for readmission' from previous studies in patients with bacteremia [56].

Cost-effectiveness of de-labelling individuals with an incorrect penicillin allergy label

Four studies reported results combining differences in costs and health benefits in cost-effectiveness analyses, all of them using decision trees. One study compared skin prick testing (SPT) with no SPT over a 1-year time horizon, including the inpatient and outpatient costs of antibiotic therapy and care for complicated infections and health-related quality of life losses associated with readmissions and adverse events [56]. A second study modelled outcomes of SPT-guided management of patients with

*S. aureus* infective endocarditis followed up until hospital discharge and found it to have lower expected costs and higher health-related quality of life (utilities) than the alternative of not testing and treating patients with vancomycin [55]. In contrast, SPT-guided vancomycin prophylaxis for cardiovascular surgery patients with a PAL had incremental costs per anaphylactic case avoided above USD 400,000 and were not recommended for routine use instead of oral history-based management [57]. The fourth study evaluated ST + OC-guided intrapartum Group B Streptococcus (GBS) prophylaxis in pregnant women,  in which the costs were included for penicillin allergy and GBS sensitivity testing and antibiotics, resulting in a cost per additional patient appropriately treated of USD 1122 relative to usual treatment according to the PAL [58].

## Discussion

Of the 36 studies included in this review, the majority met less than half the methodology quality appraisal items required to be considered a robust cost-effectiveness analysis. Most studies were cost analysis studies, focusing on antibiotic and direct healthcare costs before and after testing. Only a few studies evaluated the impact of testing and de-labelling outside a hospital setting, and most of these included small inpatient populations, were confined to single specialties (e.g. joint replacement surgery [59, 60, 61], and breast surgery [38], and typically conducted in a single centre. Most studies were conducted over a short period of time with only a few looking at impacts beyond a 1-year time period.

This review highlights several potential economic benefits of penicillin allergy testing, primarily its impact on antibiotic acquisition cost during the index episode of care. Three studies explored the impact of allergy testing on LOS, and two report associated cost reductions to the healthcare sector of 23%-48 due to LOS reductions over an acute admission alone or including repeat episodes over a 6 months period after testing. If confirmed by independent studies, this magnitude of effects on costs may offset the costs of the test, especially among low-risk patients timely screened for DPC after admission. There were no studies that reported on the patient benefits of an earlier discharge from hospital, e.g. due to switching from complex antibiotics to simpler oral penicillin administration [42, 48].

A couple of studies documented the cost of immediate and delayed reactions to the test, but there were no studies reporting the economic consequences of any potential reduction in the incidence of adverse events associated with using narrower spectrum agents due to de-labelling e.g. C*. difficile* infection. The impact of penicillin allergy

delabelling on antimicrobial acquisition costs in the community was measured in two studies; one followed children for a median of 12 months after being de-labelled at the ED [50] and the other adult inpatients 12 months after testing [36]. Neither the costs of any downstream health care service use for subsequent or relapsing episodes of infection, nor their associated health impacts were measured.

The median cost of skin testing plus oral challenge across five primary costing studies was USD 268 (range: 153, 480), which was less than the associated antibiotic acquisition cost reductions during the index hospital episode of care (which ranged between USD 367-591). Our study also summarises the reported unit costs of different testing strategies according to whether testing is led by an allergy specialist or non-allergy specialists in an inpatient or outpatient setting. Our review reflects the currently evolving recognition of the safety and feasibility of providing non-allergy specialist testing for penicillin allergy using DPC led by nurses or pharmacists [60]. Although the information presented in published studies did not always allow us to determine whether tested patients were of low, moderate or high risk, we found several reports involving high-risk populations, for example those undergoing hematopoietic stem cell transplant or the immunocompromised, where SPT and oral challenge was done by allergists. Staicu et al. [49] reported a potential saving of $152 if the testing was done by a non-allergist. A multi-test strategy of skin test plus an oral challenge was used in 18 out of 27 testing strategies, with nine of those having a three-step process (skin test, intradermal test, and an oral challenge). Skin testing alone was used in one study, and some studies estimated the cost of testing being carried out by non-allergists [32,33]. While the summarised unit costs of DPC are lower than skin test plus an oral challenge overall, one report of low-risk patients tested by nurse-led DPC plus remote doctor consultation (telemedicine) at USD 747 is above most of other estimates of skin

test plus an oral challenge reported by primary costing studies and the difference may therefore be driven by differences in the underlying costing method.

Our study adds to a previous systematic review of the costs associated with a self-reported penicillin allergy label [61], by focusing instead on studies evaluating the effect of penicillin allergy testing interventions. Like the previous review, we found that the majority of studies are observational in nature and focused on inpatient populations. Our study adds new evidence that de-labelling may reduce costs of hospital stay during the index episode of care, but the magnitude of this effect may still not fully offset the costs of penicillin allergy testing and studies that track outcomes over 3.5 years after testing may be required to capture important cost savings from hospital stays and outpatient attendances [52].

The heterogeneous findings across studies regarding the effect of penicillin allergy testing on the costs of antibiotic use partly reflects the varying quality in this literature. For example, in measuring the impact of testing, study designs vary from measuring antibiotic use in uncontrolled case series of tested patients or controlled tested cohorts. Several studies report the difference between the costs of the observed antibiotic use in de-labelled patients and the 'theoretical' amount of antibiotic that would have been used by these patients had they retained their PAL. Furthermore, some of these only measured costs for the subset of patients who are switched to a preferred penicillin or beta-lactam after testing as opposed to the whole sample of patients undergoing testing [39, 42].

There are limitations of this review. First, we did not consider studies published in languages other than English. In view of the growing awareness of the impact of penicillin allergy labels on patient and public health we are likely to miss important

emerging international evidence. Second, we only included studies that reported costs. Future reviews may seek to expand the criteria for inclusion to non-economic studies that report key outcomes, such as length of hospital stays, and apply a notional unit cost to derive more precise and informative measures of economic impact than presented here. Third, there is heterogeneity in reporting and costing methods used across studies, with some studies of ST only costing the test kit, whilst other studies accounted for the costs of staff performing the test, test kits and materials for in vivo and in vitro (intradermal) tests, cost of allergist and consultant time and capital costs of using the consultation room. We have mitigated against biases by excluding studies that did not account for the costs of consultation but observed variation across studies that may reflect differences in methods as opposed to variation in a consistent measure of unit cost of skin testing plus and an oral challenge or DPC test.

## Conclusions

There is limited evidence that penicillin allergy testing results in antibiotic costs savings, as published studies are observational, often uncontrolled and poorly reported. Whilst there is evidence of reduced antibiotic consumption in the community, little is known about the associated cost impact of de-labelling. There is emerging observational evidence to suggest significant cost savings to the health care system from reducing outpatient attendances and inpatient stays three to four years after testing. No evidence exists on the health-related quality of life impact of penicillin allergy testing. Future randomised controlled trials are required to address questions of optimal models penicillin allergy testing, with sufficiently long follow-ups and power to detect meaningful impacts to patients and national health services.

## Acknowledgements

This research was part of the ALABAMA project supported by the National Institute of Health Research Programme Grants for Applied Research (NIHR PGfAR), Grant No. RP-PG-1214-20007.

## Declarations

This work was part of the ALABAMA project, which sought to conduct a randomised controlled trial and cost-effectiveness analysis of testing for penicillin allergy. The authors declare no other competing interests.

## Supporting Information

S1 File. PRISMA checklist

S2 File. Search strategy

S3 File. Studies excluded at full text screening stage with reasons

S1 Table. Description of observational studies

S2 Table. Description of modelling studies

S3 Table. Cost elements measured in reviewed studies

## References

1. Renaudin JM, Beaudouin E, Ponvert C, et al. Severe drug-induced anaphylaxis: analysis of 333 cases recorded by the Allergy Vigilance Network from 2002 to 2010. Allergy. 2013;68:929–37.

2. Dhopeshwarkar N, Sheikh A, Doan R, Topaz M, Bates DW, Blumenthal KG, et al Drug-induced anaphylaxis documented in electronic health records. J Allergy Clin Immunol Pract. 2019;7(1):103–11.

3. Worm M, Moneret-Vautrin A, Scherer K, Lang R, Fernandez-Rivas M, Cardona V, et al First European data from the network of severe allergic reactions (NORA). Allergy. 2014;69(10):1397–404.

4. Blumenthal KG, Lu N, Zhang Y, Li Y, Walensky RP, Choi HK. Risk of meticillin resistant Staphylococcus aureus and Clostridium difficile in patients with a documented penicillin allergy: population based matched cohort study. BMJ. 2018 Jun 27;361:k2400. doi: 10.1136/bmj.k2400. PMID: 29950489; PMCID: PMC6019853.

5. West RM, Smith CJ, Pavitt SH, Butler CC, Howard P, Bates C, Savic S, Wright JM, Hewison J, Sandoe JA. 'Warning: allergic to penicillin': association between penicillin allergy status in 2.3 million NHS general practice electronic health records, antibiotic prescribing and health outcomes. Journal of Antimicrobial Chemotherapy. 2019 Jul 1;74(7):2075-82.

6. Krah NM, Jones TW, Lake J, Hersh AL. The impact of antibiotic allergy labels on antibiotic exposure, clinical outcomes, and healthcare costs: A systematic review. Infect Control Hosp Epidemiol. 2021 May;42(5):530-548. doi: 10.1017/ice.2020.1229. Epub 2020 Oct 16. PMID: 33059777.

7. Arnold A, Coventry LL, Foster MJ, Koplin JJ, Lucas M. The Burden of Self-Reported Antibiotic Allergies in Health Care and How to Address It: A Systematic Review of the Evidence, The Journal of Allergy and Clinical Immunology: In Practice, Volume 11, Issue 10, 2023, Pages 3133-3145.e3, ISSN 2213-2198,

8. Powell N, West R, Sandoe JAT, The impact of penicillin allergy de-labelling on the WHO AWaRe antibiotic categories: a retrospective cohort study, Journal of Hospital Infection, Volume 115, 2021, Pages 10-16, ISSN 0195-6701.

9. Macy E, Adkinson Jr NF. The evolution of our understanding of penicillin allergy: 1942-2022. The Journal of Allergy and Clinical Immunology: In Practice. 2023 Feb 1;11(2):405-13.

10. Rose MT, Slavin M, Trubiano J. The democratization of de-labeling: a review of direct oral challenge in adults with low-risk penicillin allergy. Expert Rev Anti Infect Ther. 2020;18(11):1143-1153. doi:10.1080/14787210.2020.1792775

11. Mustafa SS, Conn K, Ramsey A. Comparing direct challenge to penicillin skin testing for the outpatient evaluation of penicillin allergy: a randomized controlled trial. J Allergy Clin Immunol Pract. 2019;7(7):2163-2170. doi:10.1016/j.jaip.2019.05.037

12. Chua KYL, Vogrin S, Bury S, et al. The penicillin allergy delabeling program: a multicenter whole-of-hospital health services intervention and comparative effectiveness study. Clin Infect Dis. 2021;73(3):487-496. doi:10.1093/cid/ciaa653

13. Copaescu AM, Vogrin S, James F, et al. Efficacy of a Clinical Decision Rule to Enable Direct Oral Challenge in Patients With Low-Risk Penicillin Allergy: The PALACE Randomized Clinical Trial. JAMA Intern Med. 2023;183(9):944–952. doi:10.1001/jamainternmed.2023.2986

13. Lefebvre C, et al. Chapter 4: Searching for and selecting studies. In: Higgins JPT, Thomas J, Chandler J, Cumpston M, Li T, Page MJ, Welch VA (editors). Cochrane Handbook for Systematic Reviews of Interventions version 6.3 (updated February 2022). Cochrane, 2022. Available from www.training.cochrane.org/handbook.

14. Centre for Reviews Dissemination (2009). Systematic Reviews: CRD's Guidance for Undertaking Reviews in Healthcare. York, Centre for Reviews & Dissemination.

15. Page, M. J., J. E. McKenzie, P. M. Bossuyt, I. Boutron, T. C. Hoffmann, C. D. Mulrow, L. Shamseer, J. M. Tetzlaff, E. A. Akl, S. E. Brennan, R. Chou, J. Glanville, J. M. Grimshaw, A. Hrobjartsson, M. M. Lalu, T. Li, E. W. Loder, E. Mayo-Wilson, S. McDonald, L. A. McGuinness, L. A. Stewart, J. Thomas, A. C. Tricco, V. A. Welch, P. Whiting and D. Moher (2021). "The PRISMA 2020 statement: an updated guideline for reporting systematic reviews." BMJ **372**: n71.

16. McGowan J, Sampson M, Salzwedel DM, Cogo E, Foerster V, Lefebvre C. PRESS peer review of electronic search strategies: 2015 guideline statement. Journal of clinical epidemiology. 2016 Jul 1;75:40-6.

17. Ouzzani, M., H. Hammady, Z. Fedorowicz and A. Elmagarmid (2016). "Rayyan-a web and mobile app for systematic reviews." Syst Rev 5(1): 210.

18. Evers S, Goossens M, de Vet H, van Tulder M, Ament A. Criteria list for assessment of methodological quality of economic evaluations: Consensus on Health Economic Criteria. Int J Technol Assess Health Care. 2005 Spring;21(2):240-5. PMID: 15921065.

19. Husereau D, Drummond M, Petrou S, Carswell C, Moher D, Greenberg D, Augustovski F, Briggs AH, Mauskopf J, Loder E. Consolidated health economic evaluation reporting standards (CHEERS) statement. Bmj. 2013 Mar 25;346.

20. OECD, Purchasing power parities (PPP) (indicator), 2024. https://doi.org/10.1787/1290ee5a-en (accessed on 31 March 2024).

21. Ha, Jongrim, M. Ayhan Kose, and Franziska Ohnsorge. One-Stop Source: A Global Database of Inflation. Journal of International Money and Finance 2023; 137 (October): 102896. [https://www.worldbank.org/en/research/brief/inflation-database; last accessed: 07/04/2024]

22. Parikh P, Patel NC, Trogen B, Feldman E, Meadows JA. The economic implications of penicillin allergy. Ann Allergy Asthma Immunol. 2020;6:06.

23. Liu MY, Challa M, McCoul ED, Chen PG. Economic viability of penicillin allergy testing to avoid improper clindamycin surgical prophylaxis. The Laryngoscope. 2023 May;133(5):1086-91.

24. Lee GC. CORR Insights: Is Vancomycin-only Prophylaxis for Patients With Penicillin Allergy Associated With Increased Risk of Infection After Arthroplasty? Clinical Orthopaedics and Related Research. 2016;474(7):1607-9.

25. Gugkaeva Z, Crago JS, Yasnogorodsky M. Next step in antibiotic stewardship: Pharmacist-provided penicillin allergy testing. Journal of Clinical Pharmacy and Therapeutics. 2017;42(4):509-12.

26. Ferré-Ybarz L, Argente RS, Galán CG, Selvas PD, Falcó SN. Analysis of profitability in the diagnosis of allergy to beta-lactam antibiotics. Allergologia et Immunopathologia. 2015 Jul 1;43(4):369-75.

27. Blumenthal KG, Li Y, Banerji A, Yun BJ, Long AA, Walensky RP. The cost of penicillin allergy evaluation. The Journal of Allergy and Clinical Immunology: In Practice. 2018b May 1;6(3):1019-27.

28. Sobrino-García M, Muñoz-Bellido FJ, Moreno E, Macías E, Gracia-Bara MT, Laffond E, Lázaro-Sastre M, Martín-García C, de Arriba-Méndez S, Campanón-Toro MV, Gallardo-Higueras A, Dávila I. A Comprehensive Prospective Study of the Costs Associated With Evaluation of ß-Lactam Allergy. J Investig Allergol Clin Immunol. 2021 Feb 17;31(1):52-57. doi: 10.18176/jiaci.0457. Epub 2019 Oct 10. PMID: 31599727.

29. Sobrino M, Muñoz-Bellido FJ, Macías E, Lázaro-Sastre M, de Arriba-Méndez S, Dávila I. A prospective study of costs associated with the evaluation of β-lactam allergy in children. The Journal of Pediatrics. 2020 Aug 1;223:108-13.

30. Ramsey A, Mustafa SS, Holly AM, Staicu ML. Direct challenges to penicillin-based antibiotics in the inpatient setting. The Journal of Allergy and Clinical Immunology: In Practice. 2020 Jul 1;8(7):2294-301.

31. Chen JR, Tarver SA, Alvarez KS, Wei W, Khan DA. Improving aztreonam stewardship and cost through a penicillin allergy testing clinical guideline. InOpen Forum Infectious Diseases 2018 Jun (Vol. 5, No. 6, p. ofy106). US: Oxford University Press.

32. Allen HI, Gillespie P, Vazquez-Ortiz M, Murphy AW, Moylett EM. A cost-analysis of outpatient paediatric penicillin allergy de-labelling using telemedicine. Clinical & Experimental Allergy. 2021 Mar;51(3):495-8.

33. Brusco NK, Bury S, Chua KYL, Vogrin S, Holmes NE, Trubiano JA. Penicillin Allergy Delabeling Program: an exploratory economic evaluation in the Australian context. Internal Medicine Journal. 2023 Jan;53(1):74-83.

34. Jaoui A, Delalande D, Siouti S, Benoist G, Sève E, Ponvert C, Scheinmann P, Faour H, Neuraz A, Garcelon N, Delacourt C. Safety and cost effectiveness of supervised ambulatory drug provocation tests in children with mild non-immediate reactions to beta-lactams. Allergy. 2019 Dec;74(12):2482-4.

35. Borch JE, Andersen KE, Bindslev-Jensen C. The prevalence of suspected and challenge-verified penicillin allergy in a university hospital population. Basic & clinical pharmacology & toxicology. 2006 Apr;98(4):357-62.

36. du Plessis T, Walls G, Jordan A, Holland DJ. Implementation of a pharmacist-led penicillin allergy de-labelling service in a public hospital. Journal of antimicrobial chemotherapy. 2019 May 1;74(5):1438-46.

37. Englert E, Weeks A. Pharmacist-driven penicillin skin testing service for adults prescribed nonpreferred antibiotics in a community hospital. American Journal of Health-System Pharmacy. 2019 Dec 15;76(24):2060-9.

38. Fan B, Udeh B, Quinn N, Bernard SL, Grobmyer SR, Valente SA. Delabeling penicillin allergy in breast surgery patients: a cost analysis. The American Surgeon. 2020 Feb;86(2):75-8.

39. Foolad F, Berlin S, White C, Dishner E, Jiang Y, Taremi M. The impact of penicillin skin testing on aztreonam stewardship and cost savings in immunocompromised cancer patients. InOpen Forum Infectious Diseases 2019 Oct (Vol. 6, No. 10, p. ofz371). US: Oxford University Press.

40. Forrest DM, Schellenberg RR, Thien VV, King S, Anis AH, Dodek PM. Introduction of a practice guideline for penicillin skin testing improves the appropriateness of antibiotic therapy. Clinical infectious diseases. 2001 Jun 15;32(12):1685-90.

41. Harmon S, Richardson T, Simons H, Monforte S, Fanning S, Harrington K. The clinical and financial impact of a pharmacist-driven penicillin skin testing program on antimicrobial stewardship practices. Hospital pharmacy. 2020 Feb;55(1):58-63.

42. Jones BM, Bland CM. Penicillin skin testing as an antimicrobial stewardship initiative. American journal of health-system pharmacy. 2017 Feb 15;74(4):232-7.

43. Jones BM, Avramovski N, Concepcion AM, Crosby J, Bland CM. Clinical and economic outcomes of penicillin skin testing as an antimicrobial stewardship initiative in a community health system. InOpen Forum Infectious Diseases 2019 Apr (Vol. 6, No. 4, p. ofz109). US: Oxford University Press.

44. King EA, Challa S, Curtin P, Bielory L. Penicillin skin testing in hospitalized patients with β-lactam allergies: effect on antibiotic selection and cost. Annals of Allergy, Asthma & Immunology. 2016 Jul 1;117(1):67-71.

45. Li J, Shahabi-Sirjani A, Figtree M, Hoyle P, Fernando SL. Safety of direct drug provocation testing in adults with penicillin allergy and association with health and economic benefits. Annals of Allergy, Asthma & Immunology. 2019 Nov 1;123(5):468-75.

46. Macy E. Elective penicillin skin testing and amoxicillin challenge: effect on outpatient antibiotic use, cost, and clinical outcomes. Journal of allergy and clinical immunology. 1998 Aug 1;102(2):281-5.

47. Modi AR, Majhail NS, Rybicki L, Athans V, Carlstrom K, Srinivas P, Lang DM, Sobecks R, Kovacs CS. Penicillin allergy skin testing as an antibiotic stewardship

intervention reduces alternative antibiotic exposures in hematopoietic stem cell transplant recipients. Transplant Infectious Disease. 2019 Dec;21(6):e13175.

48. Rimawi RH, Cook PP, Gooch M, Kabchi B, Ashraf MS, Rimawi BH, Gebregziabher M, Siraj DS. The impact of penicillin skin testing on clinical practice and antimicrobial stewardship. Journal of hospital medicine. 2013 Jun;8(6):341-5.

49. Staicu ML, Holly AM, Conn KM, Ramsey A. The use of telemedicine for penicillin allergy skin testing. The Journal of Allergy and Clinical Immunology: In Practice. 2018 Nov 1;6(6):2033-40.

50. Vyles D, Chiu A, Routes J, Castells M, Phillips EJ, Kibicho J, Brousseau DC. Antibiotic use after removal of penicillin allergy label. Pediatrics. 2018 May 1;141(5).

51. Heil EL, Bork JT, Schmalzle SA, Kleinberg M, Kewalramani A, Gilliam BL, Buchwald UK. Implementation of an infectious disease fellow-managed penicillin allergy skin testing service. InOpen forum infectious diseases 2016 May 1 (Vol. 3, No. 3, p. ofw155). Oxford University Press.

52. Macy E, Shu YH. The effect of penicillin allergy testing on future health care utilization: a matched cohort study. The Journal of Allergy and Clinical Immunology: In Practice. 2017 May 1;5(3):705-10.

53. Sousa-Pinto B, Blumenthal KG, Macy E, Pereira AM, Azevedo LF, Delgado L, Fonseca JA. Penicillin allergy testing is cost-saving: an economic evaluation study. Clinical Infectious Diseases. 2021 Mar 15;72(6):924-38.

54. Su T, Broekhuizen BD, Verheij TJ, Rockmann H. The impact of penicillin allergy labels on antibiotic and health care use in primary care: a retrospective cohort study. Clinical and translational allergy. 2017 Dec;7:1-7.

55. Dodek P, Phillips P. Questionable history of immediate-type hypersensitivity to penicillin in staphylococcal endocarditis: treatment based on skin-test results versus empirical alternative treatment—a decision analysis. Clinical infectious diseases. 1999 Nov 1;29(5):1251-6.

56. Mattingly TJ, Meninger S, Heil EL. Penicillin skin testing in methicillin-sensitive staphylococcus aureus bacteremia: A cost-effectiveness analysis. PLoS One. 2019 Jan 7;14(1):e0210271.

57. Phillips E, Louie M, Knowles SR, Simor AE, Oh PI. Cost-effectiveness analysis of six strategies for cardiovascular surgery prophylaxis in patients labeled penicillin allergic. American journal of health-system pharmacy. 2000 Feb 15;57(4):339-45.

58. Thao V, Sharpe EE, Dholakia R, Ahn HH, Moriarty JP, Borah BJ, Gill MC, Theiler RN. Evaluating the cost-effectiveness of testing pregnant women for penicillin allergy. PloS one. 2023 Jan 20;18(1):e0280151.

59. Pagani NR, Moverman MA, Puzzitiello RN, Menendez ME, Barnes CL, Kavolus JJ. Preoperative allergy testing for patients reporting penicillin and cephalosporin allergies is cost-effective in preventing infection after total knee and hip arthroplasty. The Journal of Arthroplasty. 2021 Feb 1;36(2):700-4.

60. Lee OC, Cheng DC, Paul JL, Ross BJ, Hawkins BJ, Sherman WF. Economic burden of patient-reported penicillin allergy on total hip and total knee arthroplasty. The Journal of Arthroplasty. 2021 Sep 1;36(9):3067-72.

61. Bragg JT, Sudah SY, Moverman MA, Puzzitiello RN, Pagani NR, Menendez ME. Preoperative allergy testing for patients reporting penicillin and cephalosporin allergies is economically justified in preventing infection after total shoulder arthroplasty. Journal of Shoulder and Elbow Surgery. 2023 Jan 1;32(1):186-91.

60. Powell N, Stephens J, Kohl D, Owens R, Ahmed S, Musicha C, Upton M, Kent B, Tonkin-Crine S, Sandoe J. The effectiveness of interventions that support penicillin allergy assessment and delabeling of adult and pediatric patients by nonallergy specialists: a systematic review and meta-analysis. International Journal of Infectious Diseases. 2023 Apr 1;129:152-61.

61. Mattingly II TJ, Fulton A, Lumish RA, Williams AM, Yoon S, Yuen M, Heil EL. The cost of self-reported penicillin allergy: a systematic review. The Journal of Allergy and Clinical Immunology: In Practice. 2018 Sep 1;6(5):1649-54.

Figure Click here to access/download;Figure;Fig 1.docx

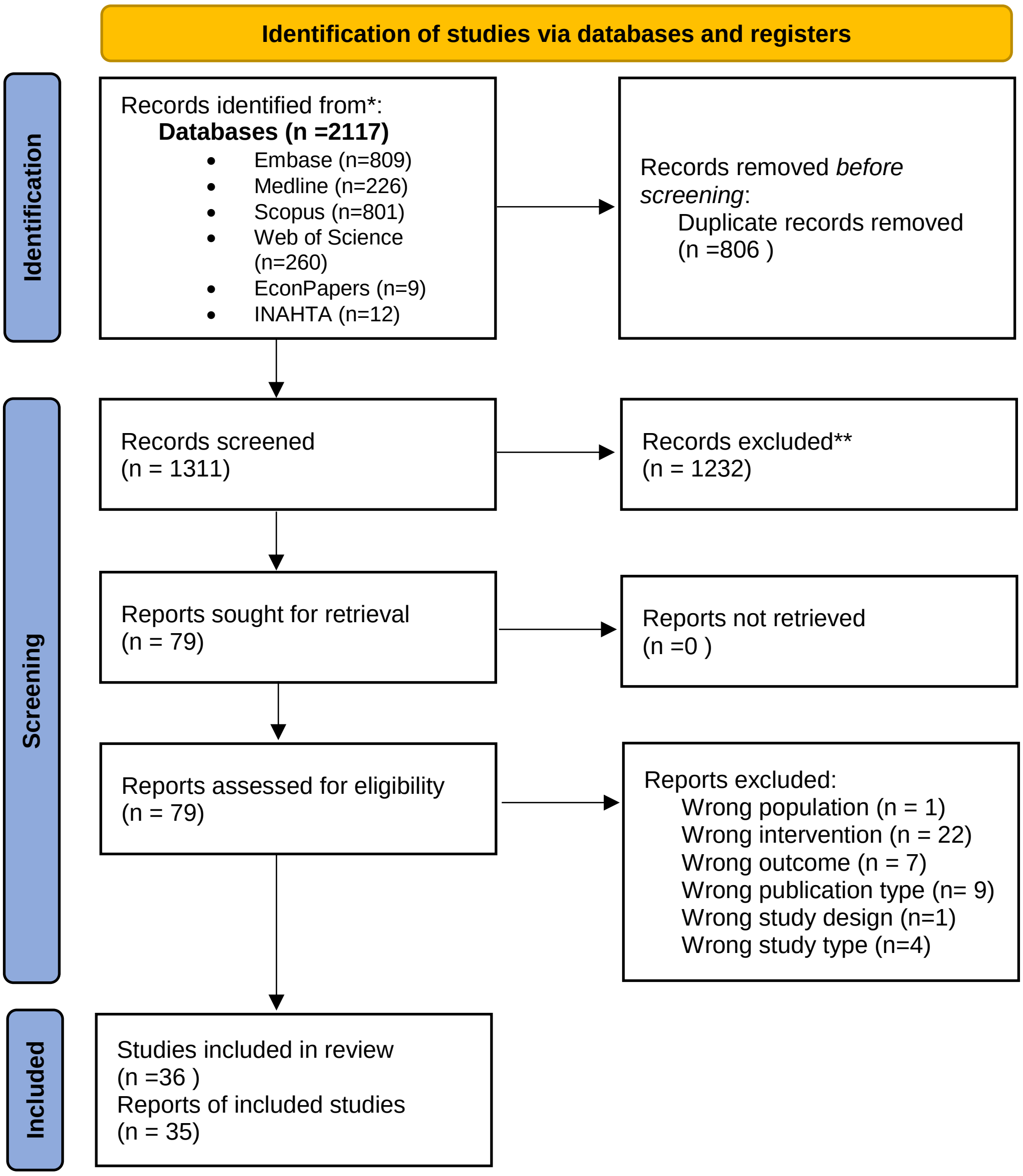


*Consider, if feasible to do so, reporting the number of records identified from each database or register searched (rather than the total number across all databases/registers).

**If automation tools were used, indicate how many records were excluded by a human and how many were excluded by automation tools.

*From:* [1]

Click here to access/download
**Supporting Information**
S1 File.docx

Click here to access/download
**Supporting Information**
S2 File.docx

Click here to access/download
**Supporting Information**
S3 File.docx

Click here to access/download
**Supporting Information**
S1 Table.docx

Click here to access/download
Supporting Information
S2 Table.docx

Click here to access/download
**Supporting Information**
S3 Table.docx
