# Supplementary material for: The cost-effectiveness of penicillin allergy testing: Evidence and gaps from a systematic review
Source: PLoS One. 2025 Dec 19;20(12):e0337131. doi: 10.1371/journal.pone.0337131 (PMC12716781; doi:10.1371/journal.pone.0337131)
Supplement: Attachment — Submitted filename: Response to reviewers 2nd resubmission.docx [file pone.0337131.s012.docx]

| No. | Comment | Response | Action |
| --- | --- | --- | --- |
| 1 | Reviewer #5  1. Introduction section: What are the gaps between this systematic review and other published systematic review articles on similar topics? What is the added value of this systematic review compared to others? (e.g. https://pubmed.ncbi.nlm.nih.gov/29355644/) | We have revised and expanded the penultimate sentence of the Introduction to address this question. | New sentence: “~~Generating e~~Evidence on the costs and benefits of different models of penicillin allergy testing services that ~~would~~ helps inform policy decisions and service design and planning is limited, as existing reviews have looked at the associated costs of penicillin allergy labels rather than assessments of testing models and predate the advent of penicillin allergy testing models for low risk patients by non-allergists [Mattingly et al. 2018].” |
| 2 | 2. Figure 1: delete this phrase "Studies included in review (n =36 )" due to the article selection flowchart, after calculating the number of excluded articles, it should be 35, not 36. 35 or 36? --> Authors need to firmly establish the inclusion and exclusion criteria for articles so that the results of the selected articles are clear and consistent. | It seems that this comment relates to the original version of Figure 1 rather than the revised and resubmitted one early this year and currently on the system. Please note that in the Fig 1.pptm file currently uploaded on to the system we have already revised the labels to read “Reports included (n=35, number of included studies 36)”. In other words, there were 35 included published articles, each but one of which reported on one evaluation; one article reported two evaluation studies. | No revisions have been made. We hope the response adequately address the question, please advise if you would think further changes are needed. |
| 3 | 3. Table 3. This table needs some tidying up especially due to some of the column headings need to be merged. | Thank you for flagging this up. Please note that the numbering refers to the original version of the manuscript rather than the revised and resubmitted version on the system. The table number in question in the resubmitted manuscript is Table 4. This table has been reformatted to merge the column heading as pointed to us by the reviewer, and the same changes have been made to Tables 5 & 6, which were added to the revised manuscript and absent from the original manuscript. | Columns merged in Tables 4-6. |
| 4 | Reviewer #6: Thank you for submitting the revised version of your manuscript.  Overall, I enjoyed reading the article, which effectively highlights the importance of a sustainable service delivery model to improve access to penicillin allergy testing and delabelling for patients who are not truly allergic. The current version provides an explanation and strengthens the earlier manuscript.  I simply have a minor comment regarding Figure 1. In its current form, the figure appears seems disorganized. | Thank you. We have put through our Fig. 1 file through the PACE pre-processing system, which has produced a tiff extension file that we have uploaded with our second resubmission. This has kept the look of the figure as originally intended. | New file has been uploaded without the figure looking as intended. |
| 5 | Journal Requirements:  Please review your reference list to ensure that it is complete and correct. If you have cited papers that have been retracted, please include the rationale for doing so in the manuscript text, or remove these references and replace them with relevant current references. Any changes to the reference list should be mentioned in the rebuttal letter that accompanies your revised manuscript. If you need to cite a retracted article, indicate the article’s retracted status in the References list and also include a citation and full reference for the retraction notice. | The reference list is complete and correct. The only change made was in the reference numbering, due to changes in response to question 1 above. | Numbering of the same references has changed. |
