# Supplementary material for: The cost-effectiveness of penicillin allergy testing: Evidence and gaps from a systematic review
Source: PLoS One. 2025 Dec 19;20(12):e0337131. doi: 10.1371/journal.pone.0337131 (PMC12716781; doi:10.1371/journal.pone.0337131)
Supplement: Attachment — Submitted filename: Response to reviewers 3rd resubmission.docx [file pone.0337131.s013.docx]

**Additional Editor Comments:**

Your manuscript presents a valuable and well-structured systematic review on the cost-effectiveness of penicillin allergy testing. Both reviewers acknowledge the overall strength and relevance of the work, noting that most substantive issues have been addressed. However, several areas still require attention to improve clarity, consistency, and presentation. Specifically, the Methods section of the abstract should be rewritten for better structure and detail; redundant text between narrative and tables should be minimized; citations must be added where missing; and language and formatting inconsistencies should be corrected for smoother readability. Minor table clarifications—such as defining “PAL” and ensuring study dates are complete—should also be addressed. Once these refinements are made, the paper will be ready for acceptance.

**Authors Response:** Thank you. We have addressed all of these comments, as described in the table below, which details the changes for each specific question raised by the reviewers.

| No. | Comment | Response | Action |
| --- | --- | --- | --- |
| 1 | Reviewer #8: |  |  |
|  | The article is well written and of good standard. Previous reviewers' comments were well addressed. I only have a minor concern. In Table 5 and 6, full meaning of PAL should be written under the tables.  Only 2 studied have dates of study in Table 1 and 2. Authors should check and clarify. | Thank you for flagging these omissions. We have implemented the corrections. | Have added the definition to Tables 5 and 6, and the study years to Table 1 and Table 2. |
|  | Reviewer #9: |  |  |
| 2 | 1. The Methods section in the abstract is poorly written and lacks structure. It is recommended to revise this section to clearly describe the review design, inclusion criteria, and evaluation approach. | We have added further detail to the Methods section of the abstract. The additional text is underlined in the next column. | “We conducted a systematic review of published economic ~~studies~~ evaluations of penicillin allergy testing in accordance with Cochrane guidelines. We searched Medline, Embase, Scopus, Web of Science, EconPapers (RePeC) and the International HTA Database (INAHTA) and included reports of full or partial economic evaluations of costs and/or health benefits of penicillin allergy testing strategies. The outcomes of interest were healthcare resource use, medical costs, and health-related quality of life for patients with a penicillin allergy label and for patients with the label removed, and cost-effectiveness. We evaluated the methodological quality of the studies using a published checklist designed for systematic reviews. The review followed a narrative synthesis.” |
| 3 | 2. There is considerable repetition between the narrative and the tables, especially in the results section. For example, Table 1 and Table 2 already list the countries of included studies, yet the same information is repeated in the “Identified records and included studies” section. It is recommended to avoid duplicating content already presented in tables. | We have deleted the list of countries of included studies in ‘Identified records and included studies section’. | Deleted text:  “Observational studies were from the US (n=17), Australia (n=3), Spain (n=3), New Zealand, Canada, Denmark, Ireland and France. Model-based evaluations related to the US (n=6); Canada (n=2) and Europe (one evaluation that reported results for US and Europe).” |
| 4 | 3. In the results section, the study quality assessment, percentages are reported without accompanying sample sizes. Please revise. | Done | The following underlined text has been added to Results, Study Quality section: Whilst most studies clearly described the population, the testing strategies under comparison and their study question, 50% (n=18) of studies adopted a time horizon that was too limited to capture relevant costs and health outcomes to their study question (i.e. until discharge for studies of inpatients or until initial antibiotic course was complete). Only 11% (n=4) and 44% (n=16) of studies adequately measured health outcomes and costs respectively (i.e. average antibiotic costs per day of regimen were reported as opposed to average per patient costs over a defined follow-up length), and 67% (n=24) clearly presented an analysis of costs and/or benefit differences. In 75% (n=27) of studies, no sensitivity analyses of assumptions used in measuring or analysing costs or benefits were presented, and 36% (n=13) of studies did not discuss the generalisability of their findings beyond their local study setting. Almost two-thirds of studies reported conflicts of interest or presented no information on whether such conflict existed (Table 3). |
| 5 | 4. Several studies are mentioned in the introduction, results and discussion sections without citation. In the Introduction, “Several recent studies have reported safe and effective testing…”. On page 83, the sentence “Only a couple of studies, both based on decision tree models, reported results in terms of quality-adjusted life years (QALYs)” it also lacks references. | Please note that the first instance referred to does include relevant citations: “Several recent studies have reported safe and effective testing for low-risk penicillin allergy labels using a direct oral challenge in children and increasingly in adults [12,13,14,15].” The same for the second passage alluded to: “Only a couple of studies, both based on decision tree models, reported results in terms of quality-adjusted life years (QALYs). One of them elicited health state utility values of treatment cure and failure in Staphylococcus aureus endocarditis under skin testing with and without adverse reaction to penicillin or toxicity from second-line therapy (vancomycin), and under no testing with and without vancomycin toxicity, from a nurse providing care for such inpatients[58]. The other was a study of skin testing in S. aureus bacteremia which obtained values for a ‘post-septic episode with no other issue’, ‘disutility for adverse event/adverse drug reaction’ independent of antimicrobial regimen and ‘disutility for readmission’ from previous studies in patients with bacteremia[60].” | No changes have been made. |
| 6 | 5. The Discussion section is generally well written and provides useful insights. However, the" Limitations section" requires thorough revision. These are specific points to consider:  On page 88, the authors list “only including studies that reported costs” as a limitation, which is misleading. This is the core objective of the review and aligns with the article’s title.  On page 88, the authors begin by listing limitations of the submitted review, then abruptly shift to critique the included studies — for example: “In addition to health-related quality of life, there is lack of evidence on long-term benefits of appropriate de-labelling to patients from avoiding delayed treatment of serious infections” — before returning to limitations of the review itself. Please revise. | Thank you for flagging these points. We have sought to address the first point by clarifying the sense of our argument.  On the second point, we have moved the paragraph with the quoted text to appear before the paragraph listing the limitations of the review on page 88. | We have addressed the first point, by adding the underlined text and deleting the crossed text in the following sentence:  “Second, we ~~only included~~ excluded studies that reported quantities of resource use, such as length of hospital say, without providing the associated costs. Future reviews may seek to expand the criteria for inclusion to non-economic studies that report key outcomes, such as length of hospital stays, and apply a notional unit cost to derive more precise and informative measures of economic impact than presented here.”  On the second point, we have moved the paragraph of the quoted text to appear before the paragraphs listing the limitations of the review. |
|  | The manuscript requires thorough language revision to improve clarity and flow. Several sections contain inconsistent sentence structure. Example - Abstract: “We conducted a systematic review of published economic studies of penicillin allergy testing.” Repetitive phrasing- “published economic studies” and “systematic review” already imply this. The Results section: “This yielded 35 unique articles reporting on 36 studies…” is confusing and needs clarification. | Thank you for your suggestions and pointers to instances of unclear sentences.  We have read the article and have made a few additions to improve flow and clarity. As presented in the next column.    On the first instance, we have rephrased the sentence as detailed in the next column.  On the second instance, we have added a clarification. | On the first instance, we have added the underlined text to and deleted the crossed text from the following sentence:  “We conducted a systematic review of published economic ~~studies~~ evaluations of penicillin allergy testing in accordance with Cochrane guidelines.”  On the second instance, the underlined text has been added:  “This yielded 35 unique articles reporting on 36 studies (unlike the rest of the articles, which reported one economic evaluation study each, one published article reported two economic evaluation studies) meeting the final inclusion criteria.”  Additional changes, include adding the underlined text:  Discussion, page 88.  “We found a number of key areas for future research. In addition to health-related quality of life, there is a lack of evidence…”  “Despite the limitations, the reviewed evidence ~~studies~~ suggests that the economic case for penicillin allergy delabelling may be better made by carefully selecting according to patients on high cost antibiotics,…”  “Our study ~~suggests~~findings are also consistent with the view that ~~more~~ efficient ~~penicillin allergy testing~~ service delivery models that rely on trained non-allergy specialist are required ~~in order~~ for ~~their~~ the adoption of penicillin allergy testing to appeal to service managers and payers.” |
